# The Choice of PCR Primers Has Great Impact on Assessments of Bacterial Community Diversity and Dynamics in a Wastewater Treatment Plant

**DOI:** 10.1371/journal.pone.0076431

**Published:** 2013-10-01

**Authors:** Nils Johan Fredriksson, Malte Hermansson, Britt-Marie Wilén

**Affiliations:** 1 Department of Civil and Environmental Engineering, Water Environment Technology, Chalmers University of Technology, Gothenburg, Sweden; 2 Department of Chemistry and Molecular Biology, University of Gothenburg, Gothenburg, Sweden; International Atomic Energy Agency, Austria

## Abstract

Assessments of bacterial community diversity and dynamics are fundamental for the understanding of microbial ecology as well as biotechnological applications. We show that the choice of PCR primers has great impact on the results of analyses of diversity and dynamics using gene libraries and DNA fingerprinting. Two universal primer pairs targeting the 16S rRNA gene, 27F&1492R and 63F&M1387R, were compared and evaluated by analyzing the bacterial community in the activated sludge of a large-scale wastewater treatment plant. The two primer pairs targeted distinct parts of the bacterial community, none encompassing the other, both with similar richness. Had only one primer pair been used, very different conclusions had been drawn regarding dominant phylogenetic and putative functional groups. With 27F&1492R, *Betaproteobacteria* would have been determined to be the dominating taxa while 63F&M1387R would have described *Alphaproteobacteria* as the most common taxa. Microscopy and fluorescence in situ hybridization analysis showed that both *Alphaproteobacteria* and *Betaproteobacteria* were abundant in the activated sludge, confirming that the two primer pairs target two different fractions of the bacterial community. Furthermore, terminal restriction fragment polymorphism analyses of a series of four activated sludge samples showed that the two primer pairs would have resulted in different conclusions about community stability and the factors contributing to changes in community composition. In conclusion, different PCR primer pairs, although considered universal, target different ranges of bacteria and will thus show the diversity and dynamics of different fractions of the bacterial community in the analyzed sample. We also show that while a database search can serve as an indicator of how universal a primer pair is, an experimental assessment is necessary to evaluate the suitability for a specific environmental sample.

## Introduction

In many environments bacterial communities are complex, with high number of individuals and high diversity. For example, estimates for bacterial communities in soil are in the range of 10^7^ -10^10^ bacterial cells [1,2] of 10^3^ -10^5^ different taxa [2,3]. It is well established that only a fraction of this immense diversity can be described by the isolation and cultivation of single bacterial species (e.g. [4]) and microbial communities are therefore studied by cultivation-independent methods, typically using PCR targeting the 16S rRNA gene.

The 16S rRNA gene has several conserved regions which are common to a large number of bacterial species, and variable regions, which are shared by fewer species. The conserved regions are used for the design of PCR primer pairs when the aim is to amplify as many bacterial species as possible. These primers are often referred to as *universal primers* implying that the target sequence is universally distributed. However, no universal primer pair can target all bacteria ( [5,6]), and different universal primer pairs may amplify different fractions of a community. Evaluations and comparisons of universal primers are therefore necessary when 16S rRNA genes are used to assess bacterial community structure.

Both fast comparisons and thorough evaluations of universal primers can be made using on-line tools such as those available through the Microbial Community Analysis (MiCA) web site [7], the SILVA ribosomal RNA gene database project [8] or the Ribosomal Database Project (RDP) [9]. For example, in an extensive study, Klindworth et al. [6] used the SILVA ribosomal RNA gene database project [8] to evaluate the overall coverage of 512 primer pairs. However, when such tools are used the analysis is based on all deposited 16S rRNA genes in a database, regardless of environmental origin. Such comparisons may not be entirely adequate to evaluate the suitability of a primer pair for a specific environment. In addition, even when specific databases are used, the specificity predicted by the database comparison can be different from the observed specificity in an actual experiment [10]. Empirical comparisons of universal primers are therefore required.

Many different universal primer pairs have been compared using samples from a range of different environments and the fact that different primer pairs amplify different fractions of a microbial community have been illustrated by differences in DNA fingerprint patterns (e.g. Sipos et al. – rhizoplane [11], Fortuna et al. - soil [12]) and composition of gene libraries (e.g. Hong et al. – marine sediments [13], Lowe et al. – pig tonsils [14]). How different the amplified fractions are is highly dependent on which primer pairs that are compared and on which environment that is sampled, i.e. the composition of the sampled community. In some studies there are only minor differences between primer pairs [15], while other studies show larger differences [13,14].

Although the choice of primer pair clearly will affect which bacterial species that are detected, it is still common practice to only use one primer pair in environmental surveys. It is accepted that the resulting description of the bacterial community is not complete and, for example, by calculating the coverage of a 16S rRNA gene library, it is estimated how representative the description is [16]. However, estimations of community richness and gene library coverage are only based on the observed taxa, i.e. the community targeted by the primer pair, and does not reveal if there are other taxa in the true community that are not targeted by the primer pair. Without an experimental evaluation of the primer pair that is used, the accuracy of the resulting data can only be assumed. However, this assumption may lead to incomplete or false conclusions when the microbial community composition data is analyzed together with environmental parameters, because factors of importance for non-targeted bacterial groups will be missed and parameters affected by these groups will not be identified. In this study we show that the fraction of bacteria that is not targeted by a universal primer pair can be non-trivial, both in terms of phylogenetic and functional groups, and that this affects the interpretation of the observed community dynamics.

An increased understanding, and ultimately management, of the microbial community composition and dynamics is regarded as fundamental for the improvement of biotechnological processes for wastewater treatment [17–20]. Wastewater treatment plants (WWTPs) and reactors can also be regarded as model systems for microbial ecology [21] and as such, be used for analyses of the formation [22], diversity [23] and dynamics [24,25] of complex microbial communities. Since the use of microbial community data from WWTPs goes far beyond mere descriptions of community composition, knowing the limitations of the methods we use for identification and diversity estimations is fundamental.

The primer pair 27F&1492R [26], or variants targeting the same regions of the 16S rRNA gene, is common in surveys of full-scale activated sludge WWTPs [23,27–30]. This primer pair was also determined by Klindworth et al. [6] to be the best primer pair for amplification of nearly full-length 16S rRNA sequences. However, it is likely that a considerable fraction of the sequences in the 16S rRNA databases have been generated with 27F&1492R, since it is one of the most common primer pairs. As pointed out by Klindworth et al. [6], this may increase the coverage of 27F&1492R compared to other, less common, primer pairs in theoretical primer evaluations. Experimental comparisons are therefore valuable to consolidate the findings of theoretical evaluations. However, we have found few experimental evaluations of the 27F&1492R primer pair. By comparison with a fluorescence in situ hybridization (FISH) analysis it was found that Gram-positive bacteria in activated sludge were not properly represented in a gene library generated using the primer pair 8F & 1492R [30], where the forward primer 8F targets the same region as 27F. A comparison has also been made between the primer pairs HK12 & HK13 (a variant of 27F&1492R) and JCR15 & JCR14 using activated sludge samples, but only minor differences in composition of the different targeted communities were found [15]. In this study we compare the primer pairs 27F&1492R and 63F&M1387R. The latter is an adjusted version of the 63F & 1387R primer pair which was previously evaluated using strains of all major bacterial groups, including Gram-positive bacteria, and was found to be more successful than the primer pair 27F & 1392R [31]. Gram-positive bacteria were also found in abundance in the analysis of an activated sludge sample where primer pair 63F & 1390R was used [32]. In addition, the 63F&M1387R primers were found to successfully amplify 16S rRNA genes from environmental samples where the primer pairs 27F & 1392R and 27F&1492R had failed [31]. The indication from these two studies that the primer pairs 63F & 1387R and 63F & 1390R successfully target bacterial groups missed by the more common primer pairs 27F&1492R and 27F & 1392R motivates a detailed comparison. Furthermore, the target sites for 63F&M1387R are both located in regions different from the target sites of 27F&1492R, which might enable amplification of sequences not targeted by the latter.

Activated sludge is particularly suitable for evaluations of methods aiming to describe bacterial diversity as it harbors complex microbial communities including a wide range of bacterial taxa (e.g. [33]). In this study we compare the composition, richness, evenness and temporal dynamics of the bacterial communities targeted by primer pairs 27F&1492R and 63F&M1387R in the activated sludge of a large-scale WWTP, using terminal restriction fragment length polymorphism (T-RFLP), FISH and sequence analysis. We show that both primer pairs miss a substantial part of different phylogenetic and functional groups in the activated sludge, resulting in different descriptions of community composition and dynamics. We also compare the two primer pairs using a general and an environment specific database showing that the results of theoretical comparisons of primer pairs do not necessarily match the results of empirical comparisons.

## Results

### Activated sludge community composition

**Figure 1:**
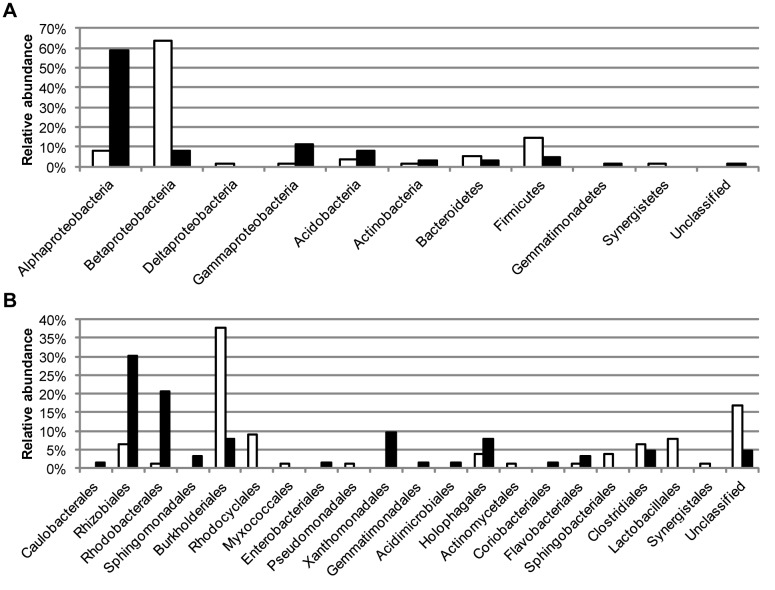
Composition of 16S rRNA gene libraries. Distribution of sequences in 16S rRNA gene libraries from an activated sludge sample generated with primer pairs 27F&1492R (77 sequences, white bars) and 63F&M1387R (63 sequences, black bars). Sequences were grouped at the level of phyla/class (panel A) and order (panel B) based on the classification by the RDP Classifier.

**Figure 2:**
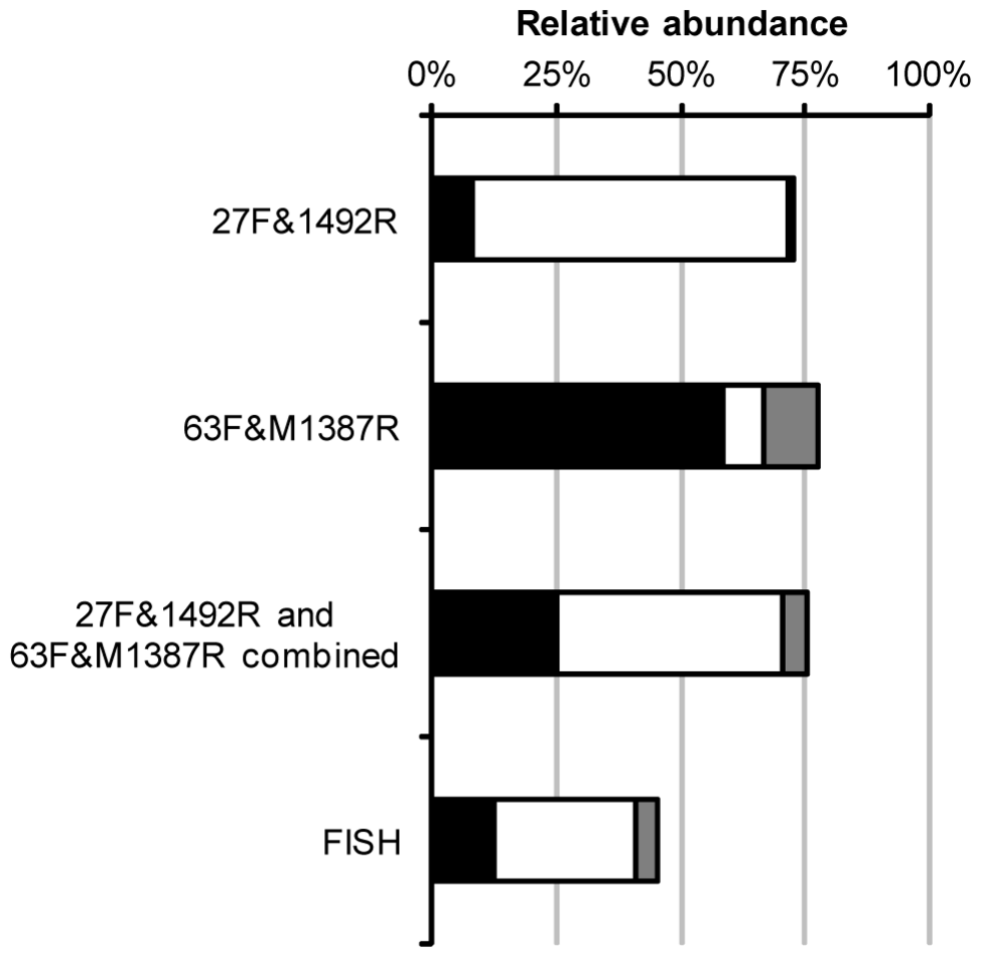
Comparison of different assessments of community composition. Comparison of the relative abundance of *Alphaproteobacteria* (black bars), *Betaproteobacteria* (white bars) and *Gammaproteobacteria* (gray bars) in an activated sludge sample. The relative abundances of the classes were derived from 16S rRNA gene libraries generated with primer pairs 27F&1492R and 63F&M1387R, analyzed separately and combined, and from FISH analysis using class-specific probes.

16S rRNA gene libraries were generated from an activated sludge sample using the primer pairs 27F&1492R and 63F&M1387R. There was a big difference in the composition between the two gene libraries (Figure 1). Sequences of class *Betaproteobacteria* dominated the 27F&1492R library while *Alphaproteobacteria* was the most frequent class in the 63F&M1387R library. There was little overlap in the communities described by the two gene libraries. A combined division of the sequences in both libraries into operational taxonomic units (OTUs) based on DNA similarities of 98.7% (species level) resulted in a total of 90 OTUs, but only 5 of these included sequences from both libraries. The sequences in the five common OTUs were only a small fraction of the total: 10% and 22% of the sequences in the 27F&1492R library and the 63F&M1387R library, respectively. The common OTUs were identified as bacteria of families *Holophagaceae* (*Acidobacteria*), *Beijerinckiaceae* (*Alphaproteobacteria*) and *Comamonadaceae* (*Betaproteobacteria*) (three OTUs). To get an overall estimation of the ratios between different taxa in the activated sludge, the number of sequences of all OTUs was related to the number of sequences in the common OTU of phyla *Acidobacteria*. With the combined data from the two primer pairs, the three most abundant taxa were *Betaproteobacteria* (45%), *Alphaproteobacteria* (25%) and *Firmicutes* (12%). As a comparison, the activated sludge sample used for gene library construction was also analyzed by FISH using probes specific for the taxa *Alphaproteobacteria*, *Betaproteobacteria* and *Gammaproteobacteria*. The combined relative abundance of *Alphaproteobacteria*, *Betaproteobacteria* and *Gammaproteobacteria* was lower in the FISH analysis, 45% compared to 73% and 78% in the 27F&1492R and 63F&M1387R library, respectively. However, the FISH analysis resulted in a ratio between *Alphaproteobacteria* and *Betaproteobacteria* of 1 to 2, equal to the ratio in the combined analysis of the clone library data (Figure 2).

LIBSHUFF [34] was used to evaluate if the two libraries were significantly different. Figure S1 shows the homologous and heterologous coverage curves for the 63F&M1387R library compared with the 27F&1492R library. The difference in shape between the curves indicates that the two samples are different. The p-value was 0.001 which means that the difference between the homologous and heterologous coverage curves was bigger for the original samples than for any of the 999 randomly generated samples. The same results (homologous and heterologous coverage curves of different shapes, p-value 0.001) were obtained for both the data set of complete and 5’ end sequences and the data set of complete and 3’ end sequences and independent if the analysis was carried out by comparing the sequences in the 63F&M1387R library with the 27F&1492R library or vice versa. The two libraries were thus determined to be significantly different.

**Figure 3:**
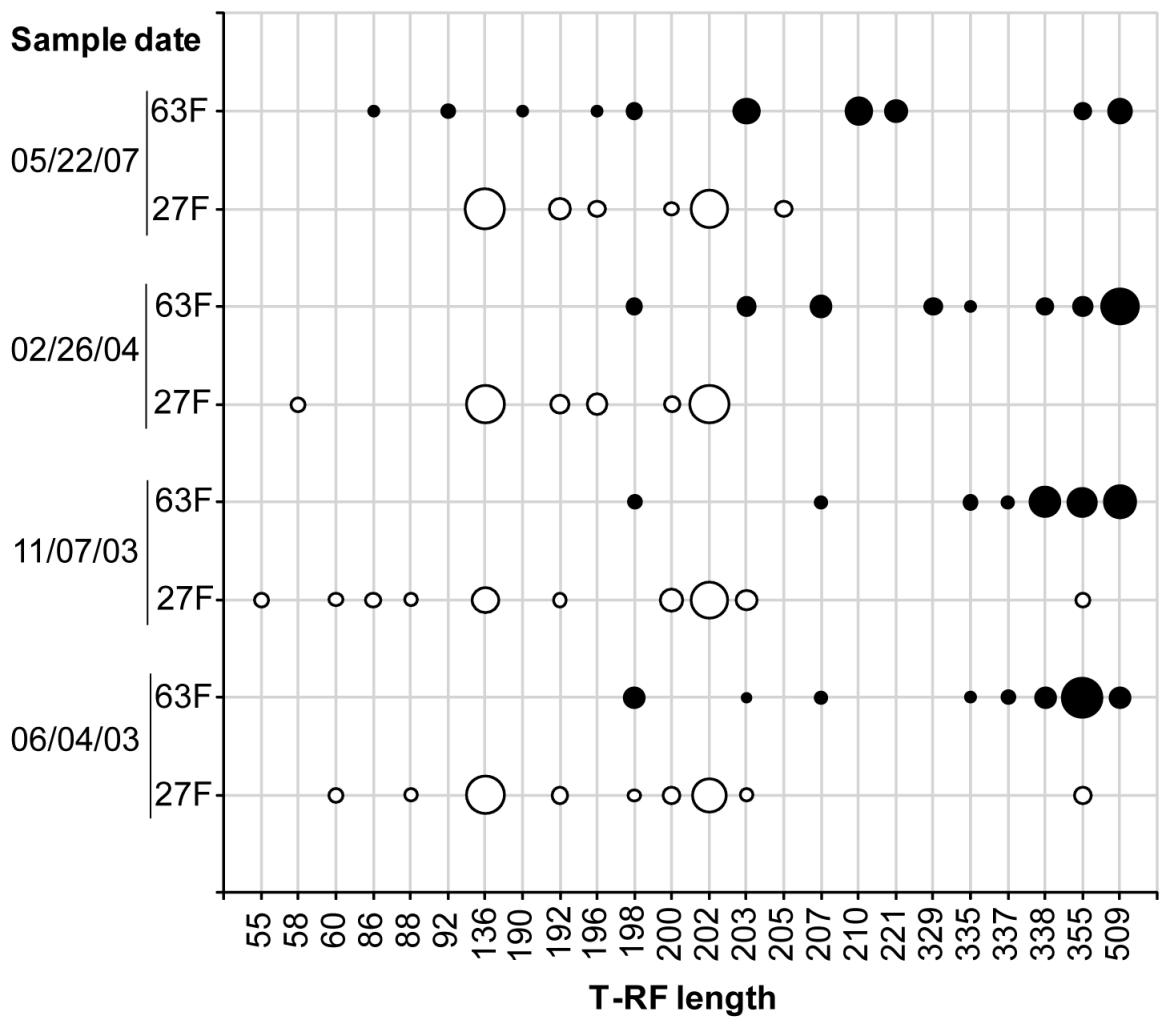
T-RFLP analysis of four activated sludge samples. T-RF profiles generated with 27F&1492R (white circles, marked as 27F on the Y-axis) and 63F&M1387R (black circles, marked as 63F on the Y-axis) using restriction enzyme *Hha*I. To allow for alignment of the T-RFs, 35 bases was added to the lengths of all T-RFs in the 63F&M1387R profiles. The size of the circles corresponds to the relative abundance of the T-RF, i.e. the peak height divided by the sum of all peak heights in the profile.

**Figure 4:**
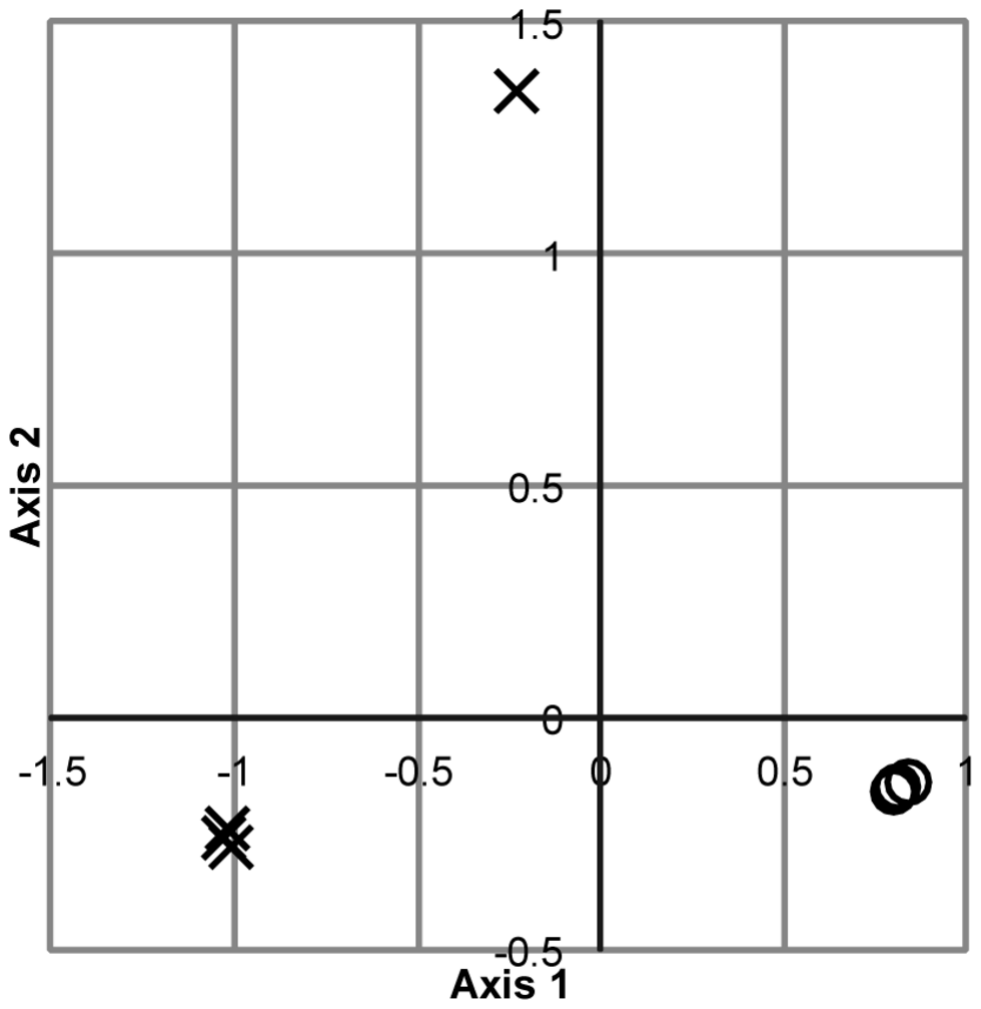
NMDS analysis of T-RF profiles. The T-RF profiles generated with 27F&1492R (circles) and 63F&M1387R (crosses) were analyzed by non-metric multidimensional scaling. The best 2-d configuration of 250 iterations is shown.

The bacterial community composition was also analyzed in four additional activated sludge samples using T-RFLP and the two primer pairs. In all four samples there were big differences between the T-RF profiles generated with the two primer pairs, with only three or less shared T-RFs per sample (Figure 3). In an ordination analysis the T-RF profiles generated with 27F&1492R clustered together, clearly separated from the T-RF profiles generated with 63F&M1387R (Figure 4). The ordination analysis also suggested that were greater differences among the 63F&M1387R profiles than among the 27F&1492R profiles, as one 63F&M1387R profile was separated from the others. To test if the differences between the profiles generated with different primers were significant a non-parametric analysis of similarity (ANOSIM) was applied. This analysis compares differences between groups, here the two groups of T-RF profiles generated with different primer pairs, with differences within groups. The test statistic R was 1, which is the highest possible value, indicating that there were differences between the T-RF profiles generated with different primer pairs. The differences were determined to be significant as the p-value was 0.026 and the hypothesis of no significant difference between the groups was rejected.

### Theoretical primer evaluations

**Figure 5:**
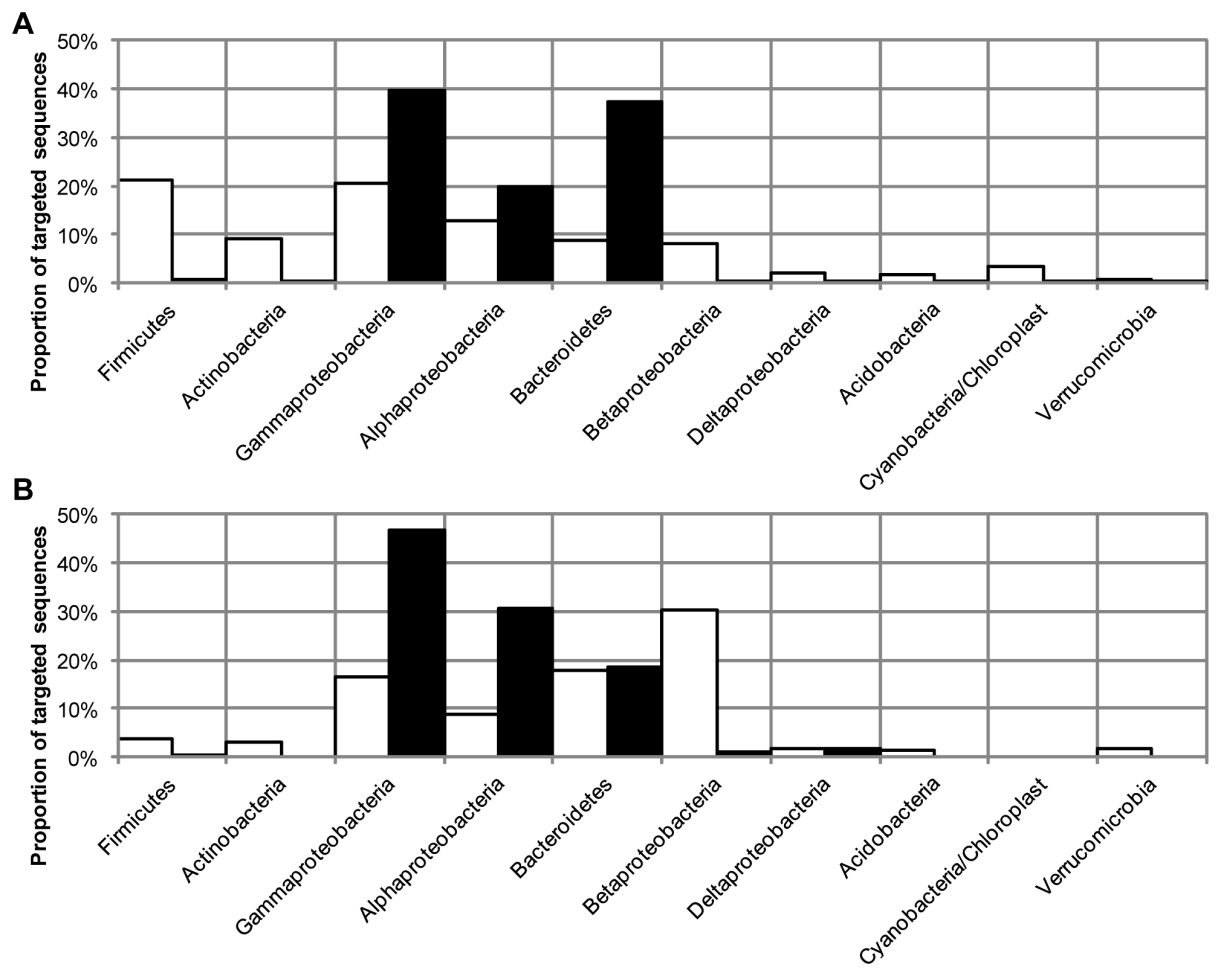
Distribution of targeted sequences in the RDP and activated sludge subset databases. Distribution of bacterial sequences in the RDP database (panel A) and in the activated sludge subset of the RDP database (panel B) targeted by 27F&1492R (white bars) and 63F&M1387R (black bars) allowing 1 mismatch between primer and target sequence. The phyla and classes included in the figure are the ten phyla and classes with the highest number of genera in the RDP database.

**Figure 6:**
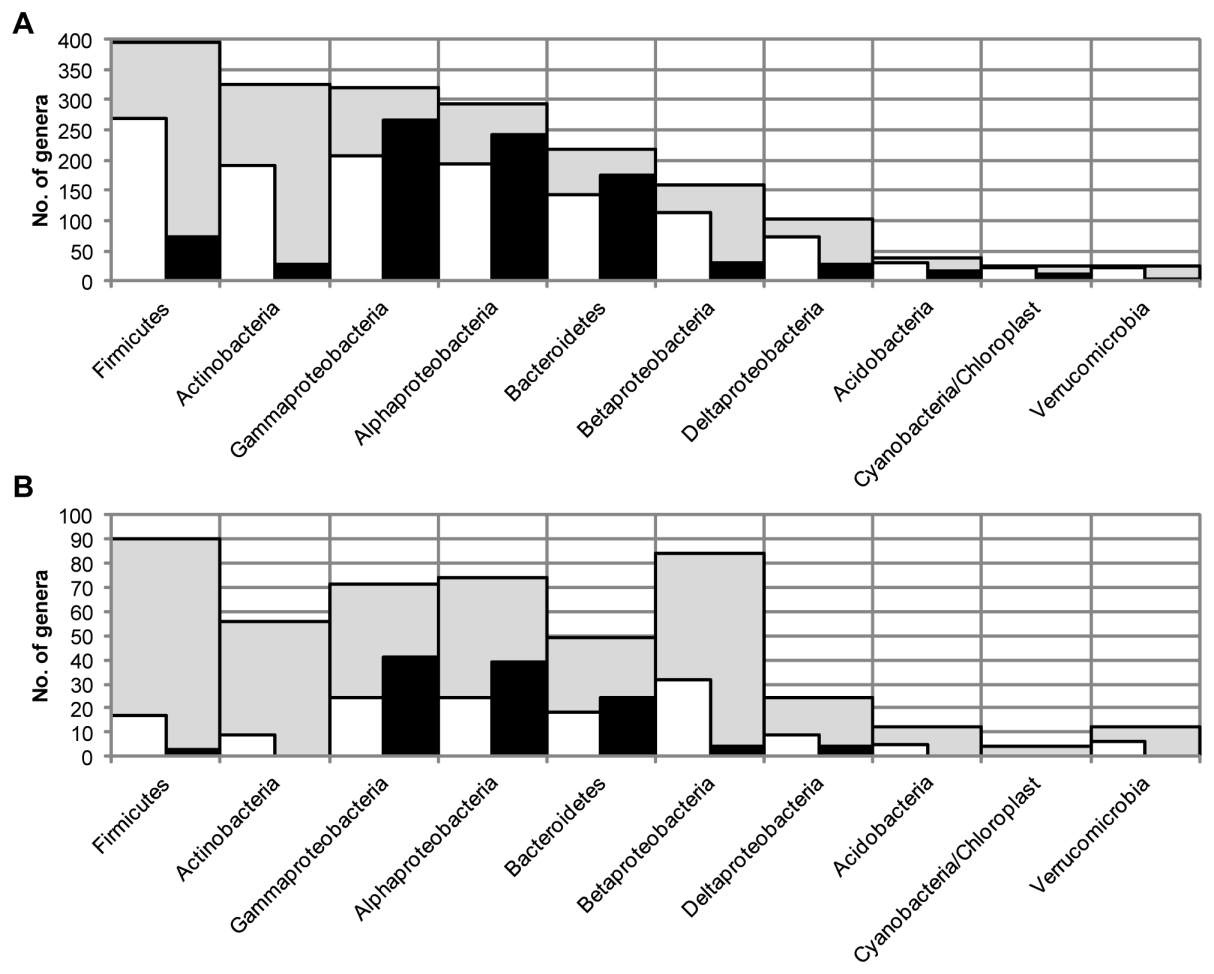
Richness of targeted phyla and classes in the RDP and activated sludge subset databases. Genus richness of different phyla and classes in the RDP database (panel A) and in the activated sludge subset of the RDP database (panel B) targeted by 27F&1492R (white bars) and 63F&M1387R (black bars) allowing 1 mismatch between primer and target sequence. The gray bars indicate the number of genera for each phylum or class in the RDP database (panel A) and in the activated sludge subset of the RDP database (panel B). The phyla and classes included in the figure are the ten richest in the RDP database.

As in the analysis of the activated sludge, there was an apparent difference in the composition and distribution of sequences in the RDP database targeted by 27F&1492R and 63F&M1387R (Figures 5 and 6). Three taxa, *Alphaproteobacteria*, *Gammaproteobacteria* and *Bacteroidetes*, make up 97% of all sequences targeted by 63F&M1387R while the sequences targeted by 27F&1492R have a more even distribution (Figure 5). The six most abundant taxa targeted by 27F&1492R: *Firmicutes*, *Gammaproteobacteria*, *Alphaproteobacteria*, *Actinobacteria*, *Bacteroidetes* and *Betaproteobacteria*, each represents between 8 and 21% of the total number of targeted sequences. The sequences targeted by 27F&1492R were also more evenly distributed in terms of number of different genera within each taxa (Figure 6). The richness of the sequences targeted by 27F&1492R was 1414 genera in 39 taxa and for 63F&M1387R the richness was 905 genera in 29 taxa. 27F&1492R covered 67% of all genera and 93% of all phyla/classes in the RDP database. The coverage by 63F&M1387R was lower: 43% of all genera and 69% of all phyla/classes in the RDP database. However, more genera of the *Alphaproteobacteria*, *Gammaproteobacteria* and *Bacteroidetes* were targeted by the 63F&M1387R primer pair than the 27F&1492R primer pair (Figure 6).

Although activated sludge contain diverse bacterial communities all taxa in the RDP database cannot be expected to be found. An activated sludge specific database was therefore generated to complement the theoretical evaluation of the two primer pairs. A search in the NCBI Nucleotide database for sequences longer than 600 bases and with any field containing the term “activated sludge” returned 12844 sequences. Most of them, 10890 sequences, were also present in the RDP database and 10878 sequences were classified as bacterial sequences. The sequences in the activated sludge subset of the RDP database (AS dataset) were distributed differently from the sequences in the complete RDP database, both in terms of number of sequences and number of genera within each taxa (Figures S2 and S3). The most common taxa in the AS dataset were, in descending order, *Betaproteobacteria*, *Gammaproteobacteria*, *Bacteroidetes*, *Alphaproteobacteria* and *Firmicutes* whereas in the complete RDP database the order was *Firmicutes*, *Gammaproteobacteria, Bacteroidetes, Actinobacteria* and *Betaproteobacteria* (Figure S2). In terms of number of genera, the three richest taxa were *Firmicutes*, *Betaproteobacteria* and *Alphaproteobacteria* in the AS dataset while *Firmicutes*, *Actinobacteria* and *Gammaproteobacteria* were the richest in the complete database (Figure S3). The total richness in the AS dataset was low as it only included 527 of 2104 genera in 30 of 42 phyla/classes present in the complete database.

The primer pairs 27F&1492R and 63F&M1387R were matched against the AS dataset. As for the searches in the complete RDP database, the distribution of the targeted sequences was different for the 27F&1492R and 63F&M1387R primer pairs (Figures 5 and 6). For 63F&M1387R, 96% of the targeted sequences were classified as *Alphaproteobacteria*, *Gammaproteobacteria* and *Bacteroidetes* (Figure 5). The sequences targeted by 27F&1492R were distributed more evenly, with the three most abundant taxa, *Betaproteobacteria*, *Bacteroidetes* and *Gammaproteobacteria*, together only representing 65% of the sequences. The richness of the sequence sets were 161 genera in 19 phyla/classes for sequences targeted by 27F&1492R while 63F&M1387R only targeted 119 genera in 10 phyla/classes. The targeted sequences included 31% and 23% of all genera and 63% and 33% of all phyla/classes in the AS dataset, for the 27F&1492R and 63F&M1387R primer pairs, respectively. As in the analysis using the RDP database, the total number of genera targeted by the 63F&M1387R primer pair was lower than for the 27F&1492R primer pair, but for some taxa, e.g. *Alphaproteobacteria*, *Gammaproteobacteria* and *Bacteroidetes*, the 63F&M1387R primer pair targeted more genera than the 27F&1492R primer pair (Figure 6). Ten phyla in the AS dataset were not covered at all by either primer, hence the low percentages of total number of phyla/classes. However, these 10 phyla only represented 1% of the total number of sequences in the AS dataset.

### Exploration and explanation of the differences in targeted taxa

**Figure 7:**
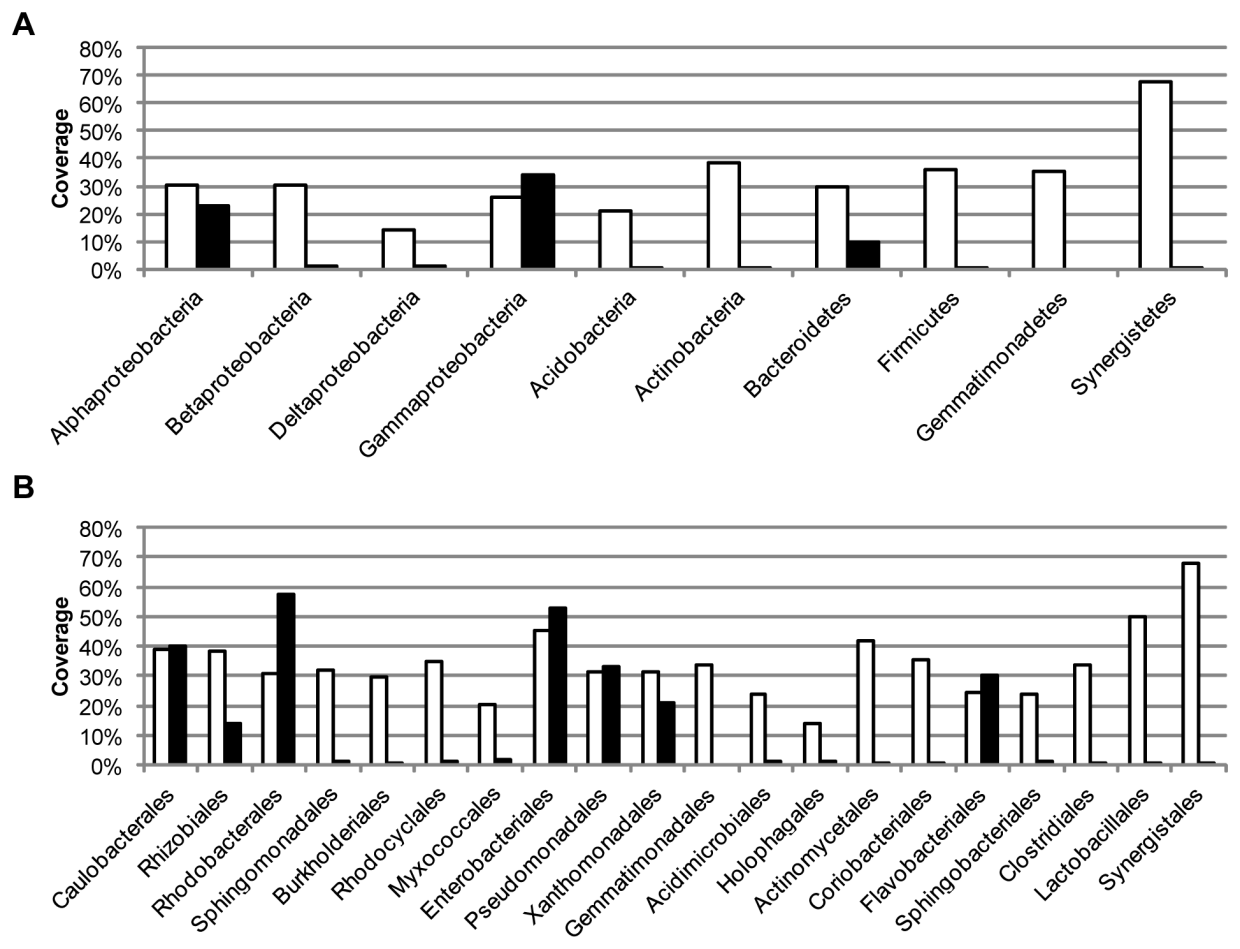
Coverage of the SILVA SSU Ref NR (release 114) database. Coverage of the SILVA SSU Ref NR (release 114) database for the primer pairs 27F&1492R (white bars) and 63F&M1387R (black bars). The coverage is the proportion of sequences long enough that match the primers with no mismatches. The coverage is shown for the phyla and taxa (panel A) and orders (panel B) that were observed in the gene libraries.

**Figure 8:**
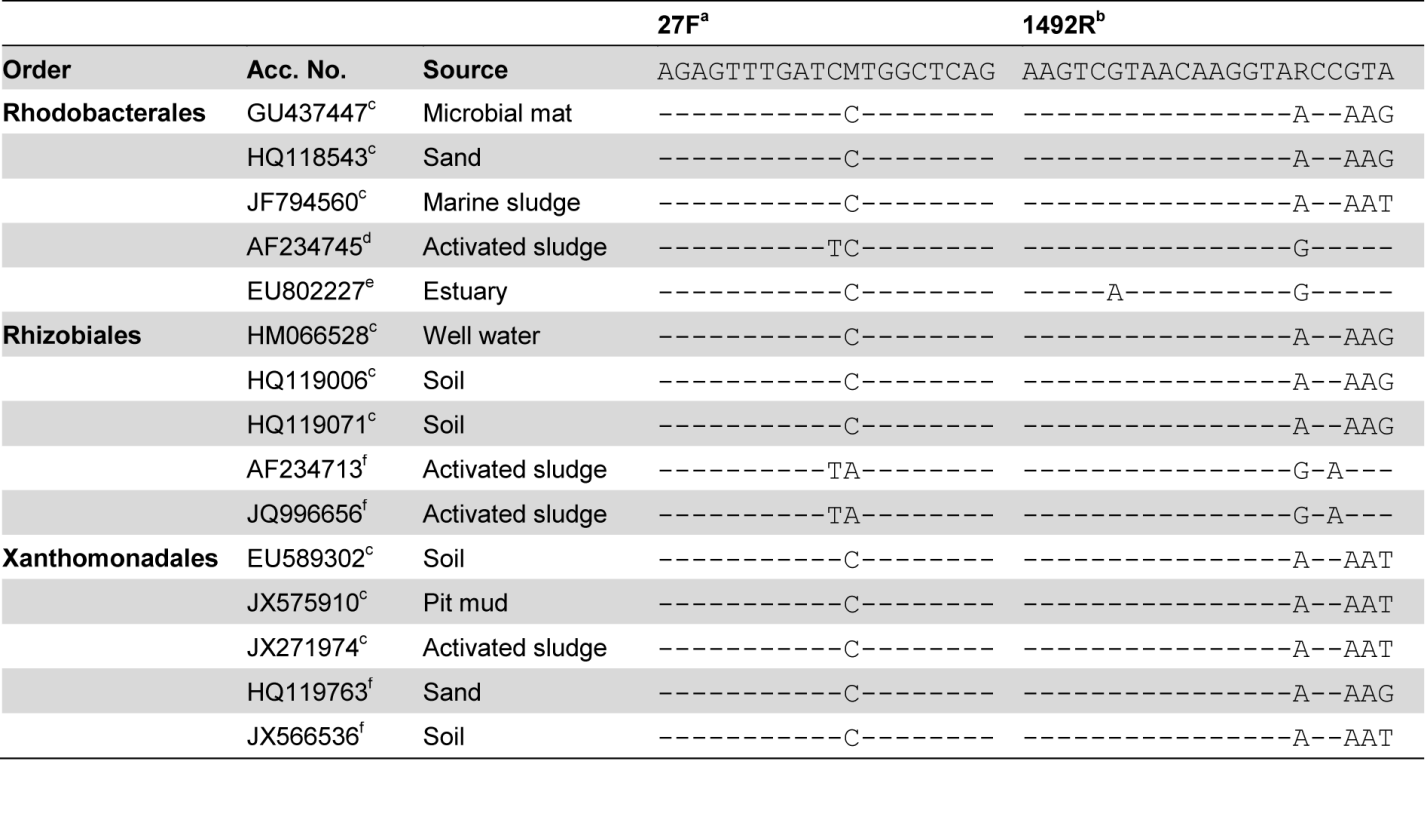
Examples of mismatches between primer pair 27F&1492R and sequences targeted by 63F&M1387R. A dash (-) indicates the same base as in the primer. a) The degenerative base M is equal to bases A and C. b) The degenerative base R is equal to bases A and G. c) Sequence in the RDP database matching the 63F&M1387R primers but not the 27F&1492R primers. d) Sequence found in the BLAST search. 98.8% sequence similarity with a sequence from the 63F&M1387R library. e) Sequence found in the BLAST search. 97.5% sequence similarity with a sequence from the 63F&M1387R library. f) Sequence found in the BLAST search. Over 99% sequence similarity with sequences from the 63F&M1387R library.

**Figure 9:**
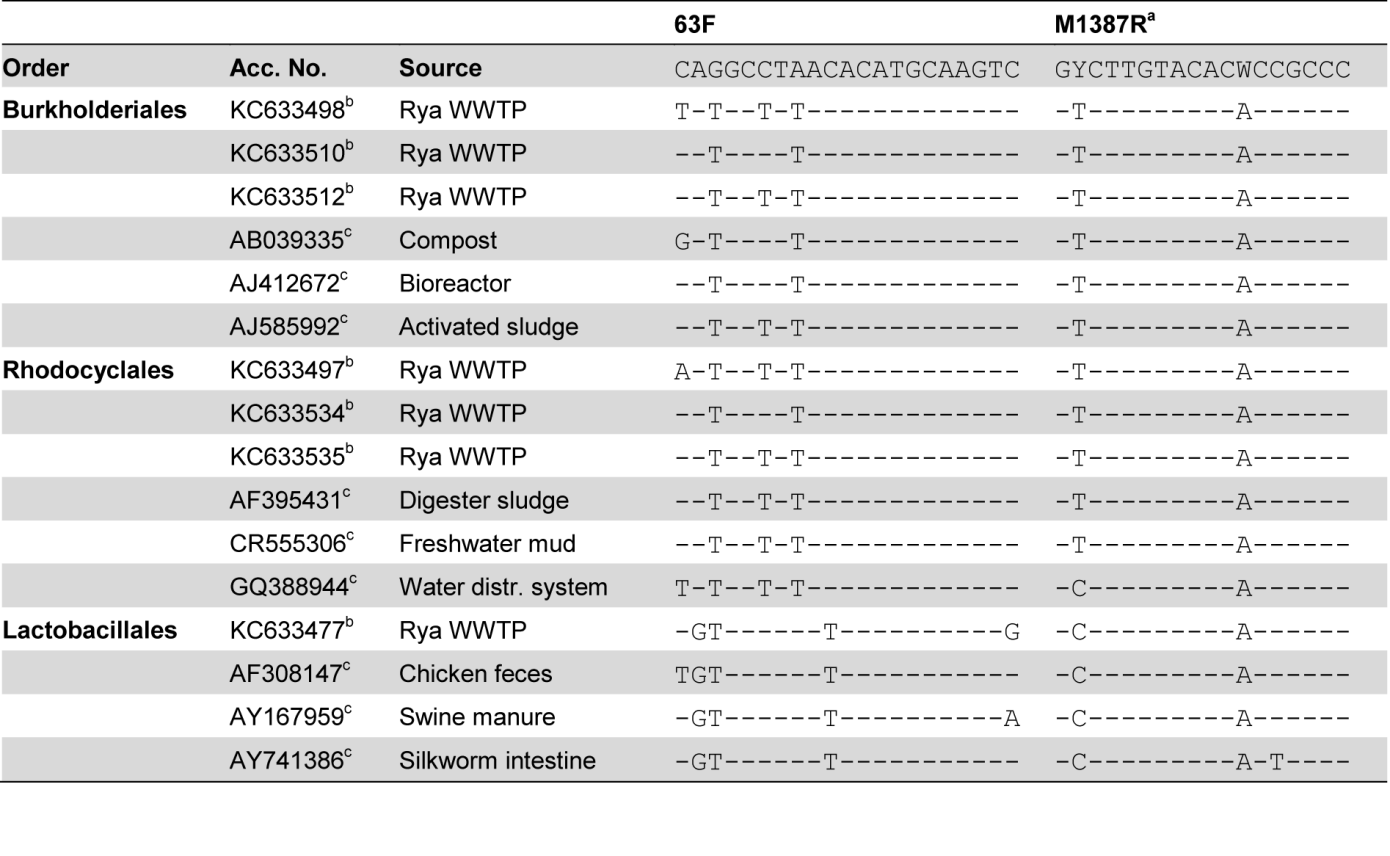
Examples of mismatches between primer pair 63F&M1387R and sequences targeted by 27F&1492R. A dash (-) indicates the same base as in the primer. a) The degenerative base Y is equal to bases C and T. The degenerative base W is equal to bases A and T. b) Sequence in the 27F&1492R gene library that does not match the 63F&M1387R primers. c) Sequence in the RDP database matching the 27F&1492R primers but not the 63F&M1387R primers.

Both in the analyzed activated sludge sample and in the database searches the two primer pairs targeted different sets of sequences. The 27F&1492R primer pair targeted more *Betaproteobacteria* and *Firmicutes* while the 63F&M1387R primer pair targeted more *Alphaproteobacteria* and *Gammaproteobacteria*. The TestPrime tool of the SILVA ribosomal RNA gene database project [8] was used to further evaluate the differences in sequence sets targeted by the two primer pairs. The coverage for the two primers, i.e. the percentage of the sequences long enough to include the primer sites that match the primers, of the taxonomic divisions observed in the gene libraries are shown in Figure 7. Overall, the 27F&1492R primer pair has a much better coverage than the 63F&M1387R primer pair. For the latter, the two classes with the best coverage, *Alphaproteobacteria* and *Gammaproteobacteria*, are also the most abundant in the gene library (Figure 1). However, at the order level, a high coverage does not correspond to a high abundance in the gene libraries. For example, 27F&1492R has a greater coverage for *Rhizobiales* sequences than 63F&M1387R, but fewer sequences were observed in the library. Likewise, 27F&1492R covers the *Xanthomonadales* sequences in the database better than 63F&M1387R, but no *Xanthomonadales* sequences were observed in the 27F&1492R library. To further explore the differences between the two primer pairs, sequences from the orders *Burkholderiales, Rhodocyclales* and *Bacillales*, which were the three most abundant in the 27F&1492R library, and from the orders *Rhodobacterales, Rhizobiales* and *Xanthomonadales*, which were the three most abundant in the 63F&M1387R library, were inspected. Very few of the sequences from the BLAST search that matched the 63F&M1387R library sequences of the orders *Rhodobacterales*, *Rhizobiales* and *Xanthomonadales* were long enough for a comparison to be made with either the 27F or the 1492R primer, let alone both of them. Among the sequences that were long enough for a comparison with the 27F&1492R primer pair there were both matching and non-matching sequences (see Figure 8 for examples of mismatches). An evaluation was also made of the sequences in the RDP database that matched the 63F&M1387R primer pair but not the 27F&1492R primer pair. Here it could be seen that the primer pair 27F&1492R does not match some sequences from the dominant orders in the 63F&M1387R library mainly due to mismatches with the 1492R primer (Figure 8). It should be noted that the analyzed sequences from the RDP database were not highly similar to the sequences in the 63F&M1387R library, the maximum similarity between the RDP sequences and the library sequences was 97.7%, 97.3% and 96.5% for *Rhodobacterales*, *Rhizobiales* and *Xanthomonadales*, respectively. However, most library sequences of the orders *Rhizobiales* and *Rhodobacterales* (11 of 19 and 8 of 13, respectively) were more similar to the RDP sequences targeted by only 63F&M1387R than to the RDP sequences targeted by both primer pairs. For *Xanthomonadales* the library sequences were equally similar to the RDP sequences targeted by only 63F&M1387R as to the RDP sequences targeted by both primers. In essence, we cannot conclude that the 27F&1492R primer pair failed to amplify more of the *Rhodobacterales, Rhizobiales* and *Xanthomonadales* in the activated sludge because of the same mismatches as seen in the RDP sequences or in the sequences from the BLAST search. However, we can see that the coverage by the 27F&1492R primer pair of these orders is not complete and based on the RDP sequences, it is mainly due to mismatches with the reverse primer. This is in contrast with the screening of all sequences in the SILVA database where the majority of the mismatches were due to mismatches with the forward primer (Table 1). The 63F&M1387R primer pair does not target sequences from the dominant orders in the 27F&1492R library almost exclusively because of mismatches with the 63F primer (Figure 9). Here too, this is different from the screening of all sequences in the SILVA database where 98% of the sequences not targeted by the primer pair had mismatches with the M1387R primer (Table 2). While 27F&1492R did target sequences in the RDP database of the *Rhodobacterales, Rhizobiales* and *Xanthomonadales* orders, including some not targeted by 63F&M1387R, 63F&M1387R only targeted a few *Burkholderiales* sequences, but no *Rhodocyclales* or *Bacillales* sequences.

**Table 1:** Distribution of sequences with mismatches with the 27F&1492R primer pair.

	**SILVA Ref NR^a^**	**Rhodobacterales^b^**	**Rhizobiales^b^**	**Xanthomonadales^b^**
**Total no.**	43561	107	62	20
**Mismatch only with 27F^c^**	64%	11%	8%	20%
**Mismatch only with 1492R^d^**	8%	73%	85%	70%
**Mismatch with both^e^**	28%	16%	7%	10%

a) Number of sequences in the SILVA SSU Ref NR (release 114) database that do not match the 27F&1492R primers. b) RDP database sequences of the given order that matched the 63F&M1387R primers but not the 27F&1492R primers. c) The proportion of the total number of analyzed sequences that only had mismatches with the 27F primer. d) The proportion of the total number of analyzed sequences that only had mismatches with the 1492R primer. e) The proportion of the total number of analyzed sequences that had mismatches with both the 27F and the 1492R primer.

**Table 2:** Distribution of sequences with mismatches with the 63F&M1387R primer pair.

	**SILVA Ref NR^a^**	**Burkholderiales^b^**	**Rhodocyclales^b^**	**Lactobacillales^b^**
**Total no.**	192488	86	23	115
**Mismatch only with 27F^c^**	2%	97%	100%	98%
**Mismatch only with 1492R^d^**	52%	0%	0%	0%
**Mismatch with both^e^**	46%	3%	0%	2%

a) Number of sequences in the SILVA SSU Ref NR (release 114) database that do not match the 63F&M1387R primers. b) RDP database sequences and gene library sequences of the given order that matched the 27F&1492R primers but not the 63F&M1387R primers. c) The proportion of the total number of analyzed sequences that only had mismatches with the 63F primer. d) The proportion of the total number of analyzed sequences that only had mismatches with the M1387R primer. e) The proportion of the total number of analyzed sequences that had mismatches with both the 63F and the M1387R primer.

### Impact of PCR primer choice on the observed diversity

**Figure 10:**
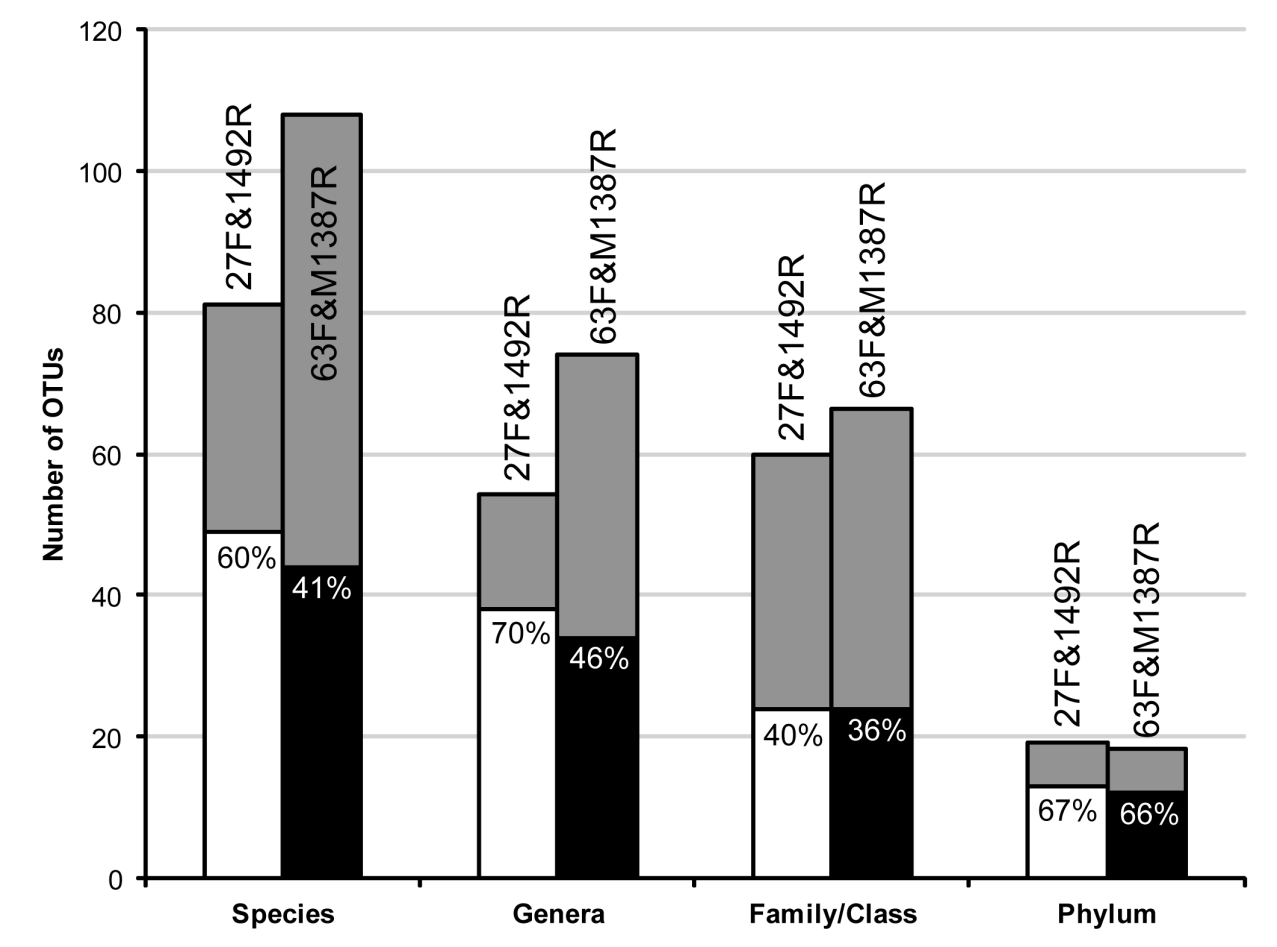
Observed and estimated richness of the 27F&1492R and 63F&M1387R 16S rRNA gene libraries. Gray columns represent richness as estimated by the Chao1 estimator. Black and white columns represent observed richness. The ratio observed: Estimated, i.e. the coverage, is given within each column. The taxonomic levels were approximated by DNA similarities of 98.7%, 95%, 90% and 80%, for species, genera, family/class and phylum, respectively.

The richness of the two gene libraries were similar, regardless if counts were based on phylogenetic classification (26 genera in 9 phyla/classes and 28 genera in 9 phyla/classes, for the 27F&1492R and 63F&M1387R library, respectively) or DNA similarities, approximating phyla and genera with 80% and 95% similarity, respectively (38 genera in 13 phyla and 34 genera in 12 phyla, for the 27F&1492R and 63F&M1387R library, respectively). However, for the 27F&1492R library the estimated richness was much lower at the level of species and genera, resulting in a greater estimated coverage (Figure 10). The sequences in the 63F&M1387R library appeared to be distributed more evenly than the sequences in the 27F&1492R library. In the evenness analysis using Pareto-Lorenz curves the Fo index was calculated to be 68% for the 63F&M1387R library and 75% for the 27F&1492R library, the lower index indicating a more even distribution (Figure S4).

### Impact of PCR primer choice on the observed community dynamics

**Figure 11:**
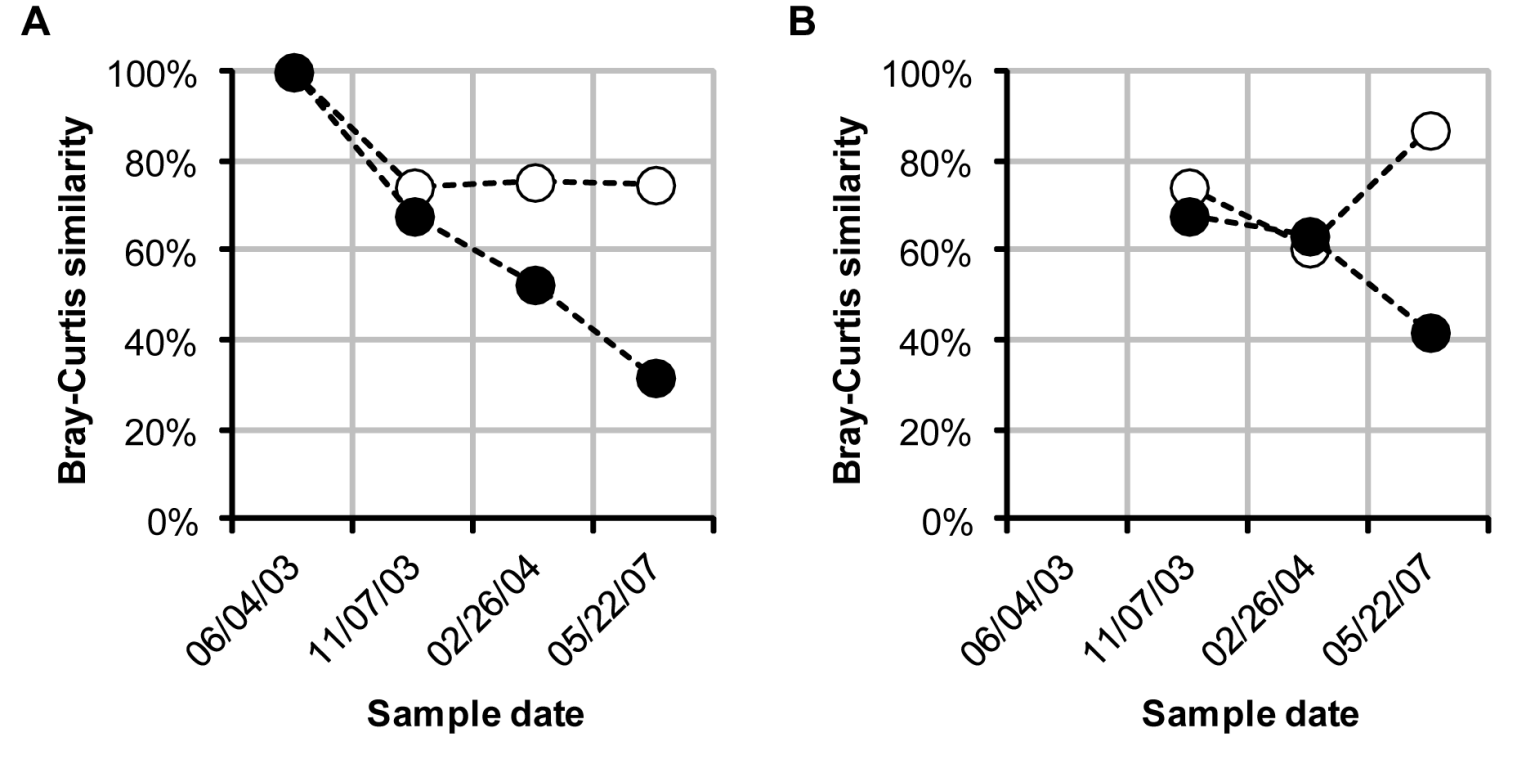
Community stability and rate of change. Community stability (panel A) Bray-Curtis similarity between the T-RF profile of 06/04/03 and all other profiles generated with 27F&1492R (white circles) and 63F&M1387R (black circles). Rate of change (panel B) Bray-Curtis similarity between subsequent T-RF profiles generated with 27F&1492R (white circles) and 63F&M1387R (black circles).

The composition of the T-RF profiles generated with 27F&1492R were found to be significantly different from the T-RF profiles generated with 63F&M1387R (Figure 3). In addition, the observed dynamics were also very different for the two primer pairs. The stability of a community over time can be analyzed by comparing all T-RF profiles with the first profile in a series of samples. While the 27F&1492R T-RF profiles showed a constant similarity of around 75% with the first T-RF profile in the series, suggesting a fairly stable community, the 63F&M1387R profiles showed a steady decrease in similarity from the first, indicating a steady deviation from the original community (Figure 11, panel A). By plotting the similarity between all consecutive T-RF profiles the times where the greatest changes in community composition occurred can be identified. The lowest similarity between two consecutive T-RF profiles was observed between November 2003 and February 2004 in the 27F&1492R analysis, while in the 63F&M1387R analysis the lowest similarity was observed between the T-RF profiles of February 2004 and May 2007 (Figure 11, panel B).

## Discussion

### The primer pairs 27F&1492R and 63F&M1387R describe different fractions of the bacterial community

The fact that primer pairs target different fractions of a community has been demonstrated in a number of studies by applying different primer pairs to a single sample [11–15]. However, the extent of primer bias and discrimination varies between different primers and environments and may be hard to predict without experimental data. The present investigation is the first one to report a significant primer bias of common universal 16S rRNA primers in the description of WWTP communities. Identification of limitations of common 16S rRNA primers is valuable because management of the diversity and dynamics of the bacterial communities in WWTPs is regarded as a possible, and perhaps even necessary, way to improve the function of the WWTPs [17–20] and to identify the factors that shape bacterial communities 16S rRNA gene sequence data is often used (e.g. [35–38]).

16S rRNA gene libraries were generated from an activated sludge sample using the two universal primer pairs 27F&1492R and 63F&M1387R, and very different descriptions of the bacterial community were obtained. Using the 27F&1492R primer pair the activated sludge community would have been described as dominated by *Betaproteobacteria* while the 63F&M1387R primer pair would have led us to believe that the activated sludge was dominated by *Alphaproteobacteria*. Different conclusions regarding the distribution of putative functional groups would also have been drawn. The sequences of the order *Rhizobiales*, the most abundant order in the 63F&M1387R library, were classified as *Beijerinckiaceae*, *Hyphomicrobiaceae* and *Methylocystaceae* which are heterotrophs [39], methylotrophs [40] and methanotrophs [41]. The *Burkholderiales* sequences, which were the most abundant in the 27F&1492R library, were almost all classified as different genera of the family *Comamonadaceae*, many of which are heterotrophs capable of denitrification [42]. Thus, with the 63F&M1387R primer pair methanotrophs and methylotrophs would have been determined to be abundant along with heterotrophic bacteria while with the 27F&1492R primer pair denitrifying heterotrophs would have been determined to be very abundant.

A previous study indicated that the primer pair 8F & 1492R [30] may fail to amplify Gram-positive bacteria in activated sludge, while another study did find Gram-positive bacteria in a gene library from activated sludge generated using 63F & 1390R [32]. In this study more Gram-positive sequences were found in the 27F&1492R library than in the 63F&M1387R library, 12% and 6% of all retrieved sequences, respectively. Of these sequences, only one from each library was of the same family, suggesting that both primer pairs do target Gram-positive bacteria, but different groups. Thus, depending on the community composition both primer pairs may appear to either fail or succeed in amplifying 16S rRNA gene sequences of Gram-positive bacteria.

There were many phyla represented in the activated sludge data set (Figure S2) that were not observed in the gene libraries. However, the phyla that were present in the gene libraries have been shown to be the most abundant in activated sludge of WWTPs and bioreactors world-wide [23,30,33]. The low number of observed phyla in the gene libraries are likely due to the relatively small library sizes. If a higher number of sequences had been analyzed less abundant phyla may also have been detected.

To further evaluate the accuracy of the descriptions of the bacterial community by the two primer pairs a comparison was made with a FISH analysis of the same activated sludge sample (Figure 2). Of course, results obtained by the FISH method may also be biased and erroneous since FISH probes, just as PCR primers, may not be as specific or inclusive as intended. Even so, the comparison can be used to highlight two aspects of the observed distribution of different taxa in the gene libraries. The comparison with the distribution obtained by FISH analysis showed that both primer pairs may overestimate the relative abundance of *Proteobacteria*, possibly because they fail to detect some other bacterial groups. However, the ratio between the *Alphaproteobacteria* and the *Betaproteobacteria*, is similar in the combined analysis of the gene library data and in the FISH analysis. This could be an indication that together, the two primer pairs describe the *Proteobacteria* accurately, at least in terms of abundance of the different classes within the phyla.

That the two primer pairs amplify distinct parts of the microbial community in the activated sludge is consistent with the results of other experimental evaluations of primer pairs. Hong et al. used marine sediment samples to compare not only two primer pairs (27F&1492R and 8F&1542R), but also two DNA extraction techniques, and found that the different methods each produced distinct results [13]. As in this study, the most abundant phyla were detected by both primer pairs, but in different proportions. Although the two primer pairs used in this study are universal in the sense that they amplified sequences from a wide range of taxa, each primer pair showed a clear bias towards certain taxa. This was also reported by Lowe et al [14] who compared gene libraries from pig tonsils generated by 27F & 1389R and 63F & 1389R. Consistent with the results of this study, the 63F primer generated a higher number of sequences of class *Gammaproteobacteria* and 27F a higher number of *Firmicutes*. These and other differences in the range of sequences targeted by the two primer pairs were also seen in the database searches (Figures 5-7). However, the results of database comparisons and theoretical evaluations of primer pairs can be misleading. In essence, it does not matter if one primer pair has a 75% coverage and another a 10% coverage of a certain taxa if the bacteria present in the sample of interest belong to the 10% that the second primer pair targeted. For example, the 27F&1492R primer pair was shown to have a greater coverage than 63F&M1387R for most orders, including the *Rhizobiales* and the *Xanthomonadales*. Despite this, sequences of these two orders were much more abundant in the 63F&M1387R library than in the 27F&1492R library. This illustrates that a high coverage of a taxa does not guarantee detection of sequences from that taxa. An evaluation of the sequences in the RDP database targeted by the two primer pairs showed that the 27F&1492R primers did match sequences of both *Rhizobiales* and *Xanthomonadales*, but that a fraction of these two orders were missed due to mismatches with the 1492R primer (Table 1). That the general conclusions from database evaluations of complete databases can differ from specific comparisons was also seen in the evaluation of the mismatches. While the majority of the mismatches between the 27F&1492R primer pair and the sequences in the SILVA SSU Ref NR (release 114) database were due to mismatches with the forward primer, the reverse was observed in the manual evaluation of three specific orders.

Based on the database searches and evaluations in this study the primer pair 27F&1492R appeared to be a better choice for assessment of bacterial diversity than 63F&M1387R since it targeted a wider range of taxa and had a much better coverage. In an extensive theoretical evaluation of primer pairs by Klindworth et al. [6], 27F&1492R was also determined to be the best choice for amplification of nearly full-length sequences. However, the experimental comparison presented here showed that for the activated sludge that was analyzed none of the two primer pairs was necessarily better than the other. Both primer pairs generated gene libraries with similar richness, both including taxa not present in the other. Thus, if only one of these two primer pairs is to be used, which of the two that is the most suitable depends on the aim of the analysis. If the focus of the analysis is on *Betaproteobacteria*, then 27F&1492R would be a better choice than 63F&M1387R since a higher number of *Betaproteobacteria* sequences was found in the 27F&1492R gene library. However, if *Alphaproteobacteria* or *Gammaproteobacteria* are of interest, 63F&M1387R would be a better choice since more sequences of these phyla were found in the 63F&M1387R gene library.

### The primer pairs 27F&1492R and 63F&M1387R describe different dynamics of the bacterial community

If two primer pairs target different fractions of a community it implicitly follows that they may also describe different community dynamics but this is rarely discussed or shown. By analyzing four activated sludge samples with the primer pairs 27F&1492R and 63F&M1387R we show that the community dynamics can be described in very different ways depending on the primer pair used. While the T-RF profiles generated with the 27F&1492R primer pair showed a fairly stable community, the 63F & M1387 T-RF profiles showed a community that steadily deviated from the initial composition (Figure 11, panel A). This result stresses that the observation of a stable bacterial community, as indicated by the 27F&1492R T-RF profiles, may be misleading.

Studies of bacterial community dynamics are often done to investigate the effect of different environmental parameters on the community composition (e.g. [24,43]). In this study we show that depending on the primer pair being used different parameters may appear to have the greatest effect. In the 27F&1492R analysis the greatest change in community composition occurred between samples two and three (collected in November 2003 and February 2004, respectively) while in the 63F&M1387R analysis the greatest change was observed between samples three and four (collected in February 2004 and May 2007, respectively) (Figure 11, panel B). Consequently, for the community targeted by 27F&1492R, changes in environmental parameters between samples two and three would seem more important than any changes occurring between samples three and four, while for the community targeted by 63F&M1387R, the T-RFLP analysis would suggest the opposite. For the four samples included in this study the primer pair 63F&M1387R detected more changes in community composition than 27F&1492R. However, differences in the described dynamics between primer pairs are likely to depend on the samples that are analyzed. As for evaluations of community composition, a primer pair that it is suitable for one set of samples may not be so for another sample set.

### Conclusions

In the present study we show that the universal 16S rRNA gene primers 27F&1492R and 63F&M1387R target different parts of the bacterial community in activated sludge samples and would have resulted in distinct conclusions regarding the structure, function and dynamics of the community. The results demonstrate that experimental comparisons of universal 16S rRNA primers can reveal differences not detected by theoretical comparisons, because while database comparisons indicated that primer pair 27F&1492R would be a better choice than 63F&M1387R, the empirical comparison showed that none of the two primer pairs was better than the other. We also conclude that different dynamics can be expected with different primers and if only one primer pair is used, which is common practice, the absence of change in the observed community composition does not necessarily indicate a stable community. Combining the results of several surveys with different universal primer pairs may therefore be necessary for a more complete description of community diversity and dynamics.

## Materials and Methods

### Ethics statement

Permission to enter the Rya WWTP and to collect activated sludge samples were granted by Gryaab AB (owner and operator of the WWTP).

### Sample collection and DNA extraction

Samples were collected at the end of the aerated basins at the Rya WWTP, a WWTP treating both industrial and municipal wastewater [44]. 50 mL of sample were centrifuged and the resulting pellet was stored at -20°C within 1.5 h from collection. DNA was extracted using Power Soil DNA Extraction Kit (MoBio Laboratories). The frozen sludge pellets were thawed, 15 mL sterile water were added and the samples were homogenized by 6 min of mixing in a BagMixer 100 MiniMix (Interscience). Water was removed by centrifugation and DNA was extracted from 0.25 g of homogenized sludge pellet according to the manufacturer’s instructions. Samples collected 06/04/03, 11/07/03, 02/26/04 and 05/22/07 were used for T-RFLP analysis. The sample collected 07/15/04 was used for generation of 16S rRNA gene libraries and FISH analysis.

### PCR for T-RFLP

16S rRNA genes were amplified using HotStarTaqPlus PCR kit (Qiagen) according to the manufacturer’s instructions. *Bacteria*-specific primer pairs used were 27F (AGAGTTTGATCMTGGCTCAG) and 1492R (TACGGYTACCTTGTTACGACTT) [26] and 63F (CAGGCCTAACACATGCAAGTC) and M1387R (GGGCGGWGTGTACAAGRC). The primer pair 63F&M1387R was based on the previously published sequences 63F and 1387R [31]. The primer 1387R has a mismatch for some bacterial sequences at position 1388 [31] and was therefore modified, which increased the number of targeted sequences in the RDP database slightly (Table S1). The primers 27F and 63F were 5’-labeled with the fluorescent dye 6 – carboxyfluorescein. PCR reactions were carried out in the provided PCR buffer with 0.5 U HotStarTaqPlus, 200µM dNTP mix, 0.1 µM of each primer and 2-5 ng DNA. The PCR started with 5 min at 95°C for Taq polymerase activation followed by 35 cycles of denaturation at 94°C for 1 min, annealing at 55°C or 60°C for the 27F&1492R and 63F&M1387R primer pairs, respectively, for 30 s and elongation at 72°C for 1 min. The reactions were ended with a final elongation step at 72°C for 7 min. To evaluate the effect of annealing temperature on the T-RF profiles PCR was also done with the primer pair 63F&M1387R and annealing temperature 55°C for the sample collected 05/22/07. Two PCR reactions were prepared for each combination of primer pair, annealing temperature and restriction enzyme.

### T-RFLP

The PCR products were purified using the Agencourt AMPure system (Beckman Coulter) and digested with 10 units of restriction enzyme *Hha*I or *Rsa*I at 37°C for at least 16 hours. The restriction digests were purified and analyzed by capillary gel electrophoresis (3730 DNA Analyzer, Applied Biosystems). The size standard LIZ1200 (Applied Biosystems) was used for fragment size determination. The software GeneMapper (Applied Biosystems) was used to quantify the electropherogram data and to generate the terminal restriction fragment (T-RF) profiles. Peaks from fragments of size 50-1020 bases with a height above 100 fluorescent units were analyzed. The total fluorescence of a sample was defined as the sum of the heights of all the peaks in the profile and was interpreted as a measure of the amount of DNA that was loaded on the capillary gel. The T-RFs of the two profiles for each primer/enzyme combination were normalized as described by Dunbar et al [45], aligned using a moving average procedure [46] and then checked manually for errors. The two profiles were combined to a single consensus profile by taking the average size, height and areas of the fragments present in both. Consensus profiles that were compared were also normalized and aligned in the same way as the two replicate profiles. To allow for comparisons of the T-RF profiles generated with 27F&1492R and 63F&M1387R, 35 bases was added to the lengths of all T-RFs in the 63F&M1387R profiles. The relative abundance of a T-RF was calculated as the peak height of that T-RF divided by the sum of all peak heights in the profile.

### Ordination analysis

Ordination analysis of all T-RF profiles was carried out using Bray-Curtis distances (described in [47]) calculated from relative abundance data. The Bray-Curtis distance coefficient is a semi-metric distance measure, i.e. not strictly metric, and therefore it cannot be used for principal coordinate analysis unless a correction for negative eigenvalues is carried out [47]. It can however be used for ordination by non-metric multidimensional scaling (NMDS). NMDS of Bray-Curtis distance matrices was carried out using the software Primer 6 (Primer-E). The analysis was performed using 250 repetitions, Kruskal stress formula number 1 and a minimum stress of 0.01.

### ANOSIM

To test if there was a significant difference between the T-RF profiles generated with 27F&1492R and 63F&M1387R, an analysis of similarity (ANOSIM) was carried out using the software PAST [48]. ANOSIM is a nonparametric multivariate procedure to test the significance of differences between groups of samples. The distances between all samples are converted to ranks and the ranks of the distances between the groups are compared with the ranks of the distances within the groups. A test statistic R is calculated which can have values between -1 and 1, where large positive values signify dissimilarity between the groups. The significance of the R-value is then calculated by Monte Carlo permutations where the samples are randomly assigned to the groups. The ANOSIM analysis was carried out using Bray-Curtis distances calculated from relative abundance data, 1000 Monte Carlo permutations and the T-RF profiles separated in two groups: profiles generated using 27F&1492R and profiles generated using 63F&M1387R.

### Cloning and sequencing

16S rRNA gene libraries were generated from an activated sludge sample collected 07/15/04. For both primer pair 27F&1492R and primer pair 63F&M1387R, 16S rRNA genes were amplified in six replicate reactions as described above, with the exception that the forward primers were not labeled. The six replicate PCR-products were pooled and purified using Qiagen QiaQuick PCR Purification Kit (Qiagen). 10 ng of purified PCR product were ligated into the plasmid vector pCR 4 TOPO (Invitrogen). One Shot DH5alpha-T1R competent *Escherichia coli* cells (Invitrogen) were transformed with the vector construct according to the manufacturer’s instructions. Transformed cells were spread on LB-agar plates with 50 µg/ml Kanamycin and incubated at 37°C for 18 hours. For each library, 96 cloned sequences were amplified directly from transformed single colonies by PCR using the vector specific primers M13forward (GTAAAACGACGGCCAG) and M13reverse (CAGGAAACAGCTATGAC). To amplify the cloned sequences, the bacterial cells were lyzed by 5 min incubation at 94°C, Taq polymerase was activated by 5 min at 95°C followed by 30 cycles of denaturation at 94°C for 45 s, annealing at 55°C for 45 s and elongation at 72°C for 1 min 45 s. The reactions were ended with a final elongation step at 72°C for 7 min. Sequencing was done using both M13forward and M13reverse as sequencing primers by Macrogen Inc. (South Korea).

### Sequence analysis

#### Sequence processing

DNA Baser (v2.91.5) was used to remove vector sequences, to trim the sequences according to quality and to assemble sequences. In the cases were the 3’ and 5’ ends of the sequences could not be assembled the partial sequences were analyzed separately. 

The sequences were checked for anomalies or chimeras in three ways: 

1-The sequences were aligned using ClustalW 1.83 [49] with default settings. The alignment was used as input to Bellerophon [50]. Sequences marked as chimeric were removed from the alignment and the remaining sequences were analyzed again. This was repeated until no chimeric sequences were detected.

2-The sequences were aligned using the greengenes web application [51] with default settings. The aligned sequences were used as input to the greengenes implementation of Bellerophon. Here each sequence is checked not only against the sequences in the clone library but also against the greengenes database of non-chimeric sequences. The similarity threshold was set to 99% and the divergence ratio was set to 1.

3-The sequences were aligned together with an *Escherichia coli* sequence (accession number U00096) using ClustalW 1.83 [49] with default settings. The alignment was then used as input to the analysis tool Mallard [52]. Sequences marked as possibly anomalous were further checked following the anomaly confirmation protocol suggested by Ashelford et al [53]. In brief, a possible anomalous sequence is analyzed together with reference sequences retrieved by BLAST using Pintail [53].

Sequences marked as chimeric or anomalous in any of the three analyses were removed.

After removal of chimeric sequences and sequences shorter than 450 bases, a total of 77 and 63 sequences were analyzed in the 27F and the 63F library, respectively. Of these 41 and 57 were near full-length assembled sequences. The remaining sequences were either only 5’-end or 3’-end sequences or 5’ and 3’-ends from the same clone that were too short to be assembled. The sequences are available in GenBank under accession numbers KC633451-KC633553 (sequences amplified by 27F&1492R) and KC633554-KC633617 (sequences amplified by 63F&M1387R).

#### Richness analysis

The non-chimeric sequences were aligned using ClustalW with default settings. Alignment of all non-chimeric sequences at the same time resulted in incorrect alignment of the 3’-end sequences and the sequences where therefore aligned in two separate sets: 1) the 5’-end sequences together with the assembled sequences, and 2) the 3’-end sequences together with the assembled sequences. In the latter the sequences were first converted to reverse complement, or anti-sense, sequences, so that they started with the reverse primer sequence. The alignments were used as input to Dnadist (the Phylip package [54]) and analyzed using the F84 distance and standard settings. The distance matrix produced by Dnadist was then converted to a similarity matrix. There were slight differences between the similarities generated from the alignment of assembled and 5’-end sequences and the similarities from the alignment of the assembled and 3-end sequences. For the assembled sequences, which were included in both data sets, the differences were due to small differences in the alignments. The unassembled 5’ and 3’-ends from the same clone showed differences in similarity with the assembled sequences, because the similarity was based on different sections of the gene (the 5’ end and 3’ end). For all clones with sequences included in both data sets, i.e. either an assembled sequence or both a 5’ and a 3’-end sequence, the similarity with the other clones was recalculated as the average similarity of the similarities from both the 5’-end alignment and the 3’-end alignment. For example: In the 5’-end alignment the 5’ –end sequence of clone D59 was determined to be 97.1% similar to the assembled sequence of clone D40 and in the 3’-end alignment the 3’-end sequence of clone D59 was determined to be 95.1% similar to clone D40. The similarity between clone D59 and D40 was then calculated to be 96.1%, the average of 97.1% and 95.1%. The similarity between a clone with only a 3’-end sequence and a clone with only a 5’-end sequence (or vice versa) was set to 0. After calculation of similarity values the clones were grouped in OTUs based on a similarity threshold of 98.7% - representing species [55], 95% - representing genus, 90% - representing family/class and 80% - representing phylum [56]. The observed frequencies of the OTUs were used as input to the program SPADE [57] and the richness of the community was estimated. The Chao1 estimator was used as a lower bound estimate of the richness.

#### LIBSHUFF

The sequences of the two gene libraries were aligned in two separate sets: 1) 5’-end alignment, including assembled sequences and 5’-end sequences, and 2) 3’-end alignment, including assembled sequences and 3’-end sequences. In the latter the sequences were first converted to reverse complement, or anti-sense, sequences, so that they started with the reverse primer sequence. The aligned sequences were analyzed using Dnadist (the Phylip package [54]) with the F84 distance and standard settings and used as input to LIBSHUFF [34]. LIBSHUFF compares two samples, or sequence libraries, by calculating differences between homologous coverage curves, and heterologous coverage curves. The coverage C is calculated by counting the number of unique sequences at a given evolutionary distance threshold D and a coverage curve is generated by calculating the coverage for a range of different evolutionary distances. To calculate the homologous coverage, C_X_, the number of unique sequences is counted by comparing each sequence with the other sequences in the same sample. To calculate the heterologous coverage, C_XY_, the number of unique sequences is counted by comparing each sequence with the sequences in the other sample. Similar homologous and heterologous coverage curves are an indication that the two samples are similar. In addition, LIBSHUFF pools the two samples and randomly separates the sequences into two new samples of the same size as the original samples. This is done 999 times and the differences between the samples in each pair of randomly generated samples are compared with the difference between the two original samples to determine if the latter is significant.

#### Classification

The sequences were classified using the RDP classifier [58]. For additional identification the sequences in the gene libraries were compared with sequences in GenBank using BLAST [59]. The BLAST searches were done 11/12/2012 and 11/13/2012.

#### Clone library comparisons and combinations

To compare the two libraries the non-chimeric sequences from both clone libraries were aligned together and analyzed and divided into OTUs as described above. To combine the two libraries and get overall ratios of different taxa and phylogenetic groups, the number of sequences of all OTUs (at OTU division threshold 98.7%) was related to the number of sequences in the common OTU of phyla *Acidobacteria*. For the OTUs of *Alphaproteobacteria* and *Betaproteobacteria* that were common to both libraries the average of the new ratios was used.

The complementarities of the sequences in the 27F1492R library with the 63F and M1387R primers were also analyzed. Only assembled sequences and sequences with both the 5’ and 3’-ends, with sequence data starting before the 63F site and ending after the M1387R site were evaluated (see Material S1 for results).

#### Pareto-Lorenz evenness curves

A Pareto-Lorenz evenness curve (see for example 60,61 for explanation and usage) was used to illustrate and quantify the evenness of the different sequence sets. The sequences were divided in OTUs based on phyla and the *Proteobacteria* classes and the OTUs were ranked from high to low, based on their abundance. The cumulative proportion of OTU abundances (Y) was then plotted against the cumulative proportion of OTUs (X) resulting in a concave curve starting at (X, Y) = (0%, 0%) and ending in (X, Y) = (100%, 100%). The functional organization (Fo) index is the horizontal y-axis projection on the intercept with the vertical 20% x-axis line, i.e. the combined relative abundance of 20% of the OTUs. In a community with high evenness all or most OTUs are equally abundant which results in a Pareto-Lorenz curve close to a straight line of 45°. The Fo index for such a community is close to 20%. Specialized communities with one or a few dominating OTUs generate concave curves with high Fo indices.

### Complete RDP database search

The RDP tool Probe Match ( [62], accessed 09/28/12) was used to compare the primer pairs 27F&1492R, 63F & 1387R and 63F&M1387R. The search was restricted to the domain *Bacteria* and but with no restriction on region, i.e. sequences of all lengths were searched. The resulting dataset was refined using the following dataset options: Both type and non type strains, both uncultured and isolates, both sequences longer and shorter than 1200 bases and only good sequences (low quality sequences were removed). The total number of sequences included in the search and the number of matches allowing 0, 1, 2 and 3 mismatches were noted. For each number of allowed mismatches (0, 1, 2 and 3), the following procedure was carried out:

I) A list of the targeted sequences was downloaded as a text file.
II) From the text files, the RDP IDs were extracted and a list of the RDP IDs were saved as a new text file.
III) Lists with the combinations (intersection, complement, unique) of the RDP ID lists for the different primers were constructed using a Perl script with the Compare::List module (available from corresponding author). The number of sequences in each of the combination lists was noted.

Subsequently, for the datasets generated by allowing 1 mismatch, the following procedure was carried out:

I) The RDP ID lists were uploaded to Sequence Cart in RDP and the corresponding sequences were retrieved.
II) The sequences in Sequence Cart were classified in RDP Classifier.
III) The hierarchy and the list of sequences from the RDP classifier was downloaded at confidence level 95%.

### Generation of a dataset with only activated sludge sequences

A search in the NCBI Nucleotide database (http://www.ncbi.nlm.nih.gov/nucleotide, accessed 10/03/12) was done using the search term (*(600:2000[Sequence Length*])* AND "activated sludge"*). The search result was saved as a list of accession numbers and uploaded to RDP Sequence Cart. The resulting dataset of retrieved sequences could not be refined like the datasets generated by Probe Match and thus included sequences of low quality. The retrieved sequences were classified using RDP Classifier, and the hierarchy was downloaded at confidence level 95%. The list of RDP IDs of the activated sludge sequences was compared with the lists of the sequences in the complete RDP database matching primer pairs 27F&1492R and 63F&M1387R as described above.

### Evaluation of the primers using the SILVA TestPrime tool

The tool TestPrime, version 1.0, (http://www.arb-silva.de/search/testprime/, accessed 06/02/2013) which is a part of the SILVA ribosomal RNA gene database project [8], was used to evaluate the primer pairs 27F&1492R and 63F&M1387R. The SSU Ref NR database (release 114) was used allowing no mismatches.

### Inspection of sequences and evaluation of mismatches

The sequences retrieved in the BLAST search that was used for classification were evaluated. Sequences that were more than 97% similar to a sequence of the order *Rhodobacterales, Rhizobiales* or *Xanthomonadales* from the 63F&M1387R library were considered, and if long enough, used for comparison with the 27F&1492R primer pair. The primer sites were located manually using the software BioEdit [63] and the mismatches were identified.

The RDP Probe Match tool and RDP Classifier were used a second time to retrieve sequences that were targeted by only one of the primer pairs or both (database accessed 06/02/2013). For *Burkholderiales, Rhodocyclales* and *Bacillales*, sequences only targeted by 27F&1492R and for *Rhodobacterales, Rhizobiales* and *Xanthomonadales*, sequences only targeted by 63F&M1387R. The same procedure as above was used but the search was restricted to the domain *Bacteria* and sequences with data from *E. coli* position 6 to 1515. No mismatches were allowed and all sequences were included (both type and non type strains, both uncultured and isolates, both sequences longer and shorter than 1200 bases and both high and low quality sequences). After retrieval of accession numbers using RDP and the procedure described above, the sequences were obtained from the Nucleotide database (http://www.ncbi.nlm.nih.gov/nucleotide/, accessed 06/02/2013).

The primer sites were located manually using the software BioEdit [63] and the mismatches were identified. For the sequences from the 27F&1492R library, the same mismatches were seen as in the RDP database sequences. However, the sequences in the 63F&M1387R library are too short to include the target sites of the primer pair 27F&1492R and the same comparison could not be made. An analysis was carried out to determine if the 63F&M1387R library sequences were more similar to the RDP sequences that were targeted only by 63F&M1387R or to the sequences targeted by both 63F&M1387R and 27F&1492R. The sequences of the orders *Rhodobacterales, Rhizobiales* and *Xanthomonadales* matching either only 63F&M1387R or both 63F&M1387R and 27F&1492R were retrieved as described above. For each order, the two sets of sequences were aligned with the 63F&M1387R library sequences from that order using ClustalW with standard settings. The aligned sequences were then analyzed using Dnadist (the Phylip package [54]) with the F84 distance and standard settings to obtain the similarities between the sequences.

### Fluorescence in situ hybridization

Activated sludge samples were fixed in 4% paraformaldehyde as previously described [64]. After fixation, 5 mL of sample were filtered onto 0.2 µm pore size membrane filter and washed with 1X PBS directly onto the filter placed in the filter holder. The samples were hybridized as described by Amann [65] for 1.5 h. Oligonucleotide probes were synthesized and 5' labeled with the fluorochrome fluorescein isothiocyanate (FITC) or one of the sulfoindocyanine dyes Cy3 and Cy5 (Thermohybaid Interactiva, Ulm, Germany). The supernatant samples were hybridized directly on the filter. All bacteria were detected by hybridizing with a mixture of EUB338, EUB338 II and EUB338 III (called EUBMIX) [66,67]. *Alphaproteobacteria*, *Betaproteobacteria* and *Gammaproteobacteria* were detected by the probes ALF1b, BET42a and GAM42a [64]. The FISH slides were viewed with a BioRad Radiance 2000 CLSM equipped with 60x inverted objective (oil immersion Nikon Eclipse TE300 Corp, Tokyo, Japan). Excitation of FITC, Cy3 and Cy5 were done at 488 nm (Ar laser), 543 nm (HeNe laser) and 637 nm (red diode laser), respectively. Emissions were collected with filters 515-530 nm BP(HQ) for FITC, 590-570 nm BP(HQ) for Cy3 and 660 nm LP for Cy5. The collected images were finally processed using Adobe Photoshop (Adobe Systems Inc., USA). For quantification at least 10 z-series with 1µm (6-24 sections) steps at no zoom applied were made for each sludge suspension sample and at least 10 images were taken of each supernatant sample on filters. The surface coverage of probe-positive cells was analyzed with the software COMSTAT [68] with the threshold set manually. The percentage coverage in relation to cells binding to EUBMIX was calculated and used as an estimate of the relative abundance of the probe-defined bacterial groups.

## Supporting Information

Figure S1 **Comparison of the gene libraries using LIBSHUFF.** Homologous (empty circles) and heterologous (filled circles) coverage curves for the 63F&M1387R library compared with the 27F&1492R library. The data analyzed was the assembled and 5’ end partial sequences. Solid lines indicate the values of (C_X_ −C_XY_)^2^ (i.e. a measure of the difference between the homologous and heterologous coverage) for the original samples and broken lines indicate the values of (C_X_ −C_XY_)^2^ for the randomly generated sample that was ranked as having the 50th greatest difference between the homologous and heterologous coverage (corresponding to p = 0.05).
(TIF)

Figure S2 **Composition of the sequence databases.** Distribution of 2 324 034 sequences in the RDP database (dark gray bars) and 10878 bacterial sequences in the activated sludge subset of the RDP database (Light gray bars). *Phylum or class with a proportion at least twice as large in one of the datasets than in the other.
(TIF)

Figure S3 **Genus richness of the sequence databases.** Number of genera within each taxa expressed as the proportion of the total number of genera in the RDP database (dark gray bars) and in the activated sludge subset of the RDP database (Light gray bars). *Phylum or class with a proportion at least twice as large in one of the datasets than in the other.
(TIF)

Figure S4 **Evenness of the gene libraries.** Pareto-Lorenz evenness curves of the 16S rRNA gene libraries generated using 27F&1492R (white circles) and 63F&M1387R (black circles). The sequences were divided in OTUs by phyla (including the *Proteobacteria* classes), as determined by classification. The Fo index for each sequence set is given.
(TIF)

Material S1 **Comments regarding annealing temperature and library size.** A supporting discussion about the impact of PCR annealing temperature and library size on the observed difference in composition between the gene library generated with the primer pair 27F&1492R and the library generated with 63F&M1387R.
(PDF)

Table S1 **Number of 2 441 787 high quality sequences in the RDP database matching different primers.**
(PDF)
