# Supplementary figures and images for: The Choice of PCR Primers Has Great Impact on Assessments of Bacterial Community Diversity and Dynamics in a Wastewater Treatment Plant
Source: PLoS One. 2013 Oct 1;8(10):e76431. doi: 10.1371/journal.pone.0076431 (PMC3788133; doi:10.1371/journal.pone.0076431)

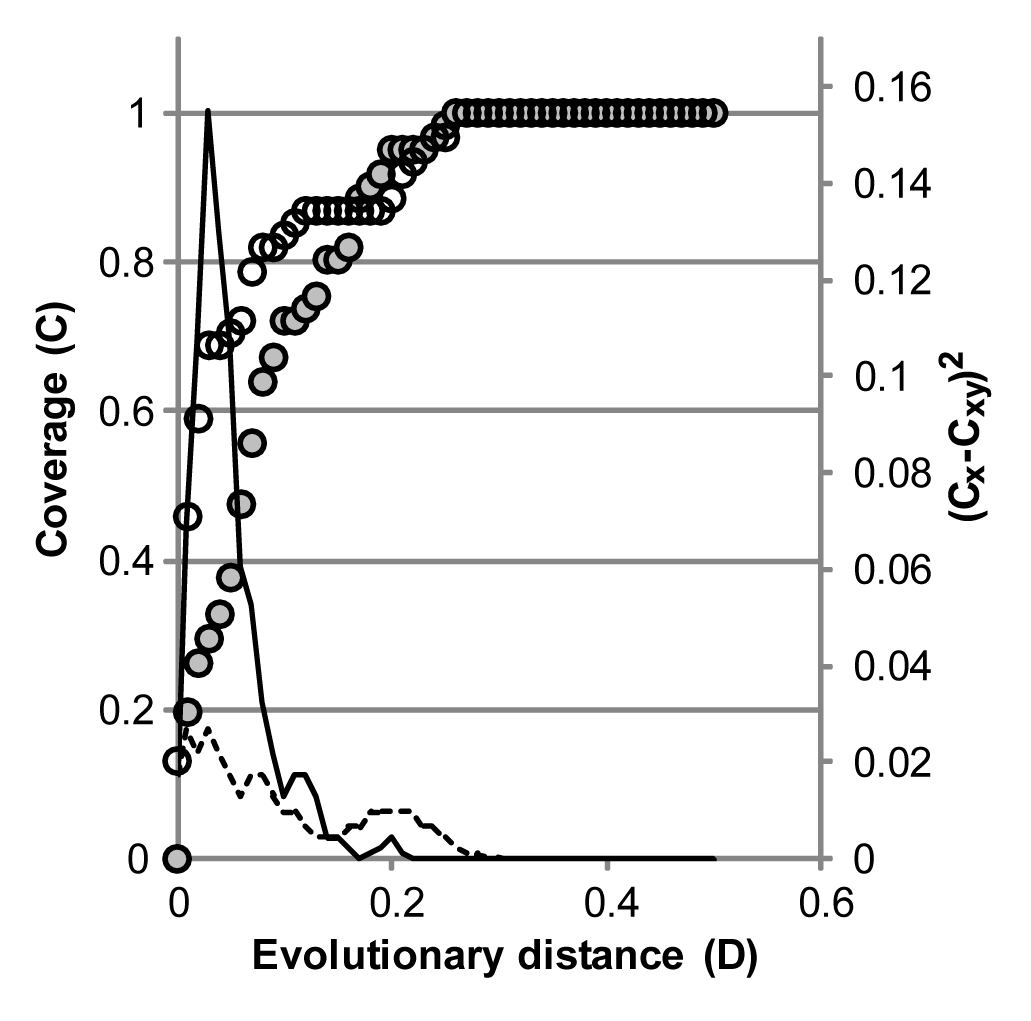

Supplement: Figure S1 — Comparison of the gene libraries using LIBSHUFF. Homologous (empty circles) and heterologous (filled circles) coverage curves for the 63F&M1387R library compared with the 27F&1492R library. The data analyzed was the assembled and 5’ end partial sequences. Solid lines indicate the values of (CX −CXY)2 (i.e. a measure of the difference between the homologous and heterologous coverage) for the original samples and broken lines indicate the values of (CX −CXY)2 for the randomly generated sample that was ranked as having the 50th greatest difference between the homologous and heterologous coverage (corresponding to p = 0.05). (TIF) [file pone.0076431.s001.tif]

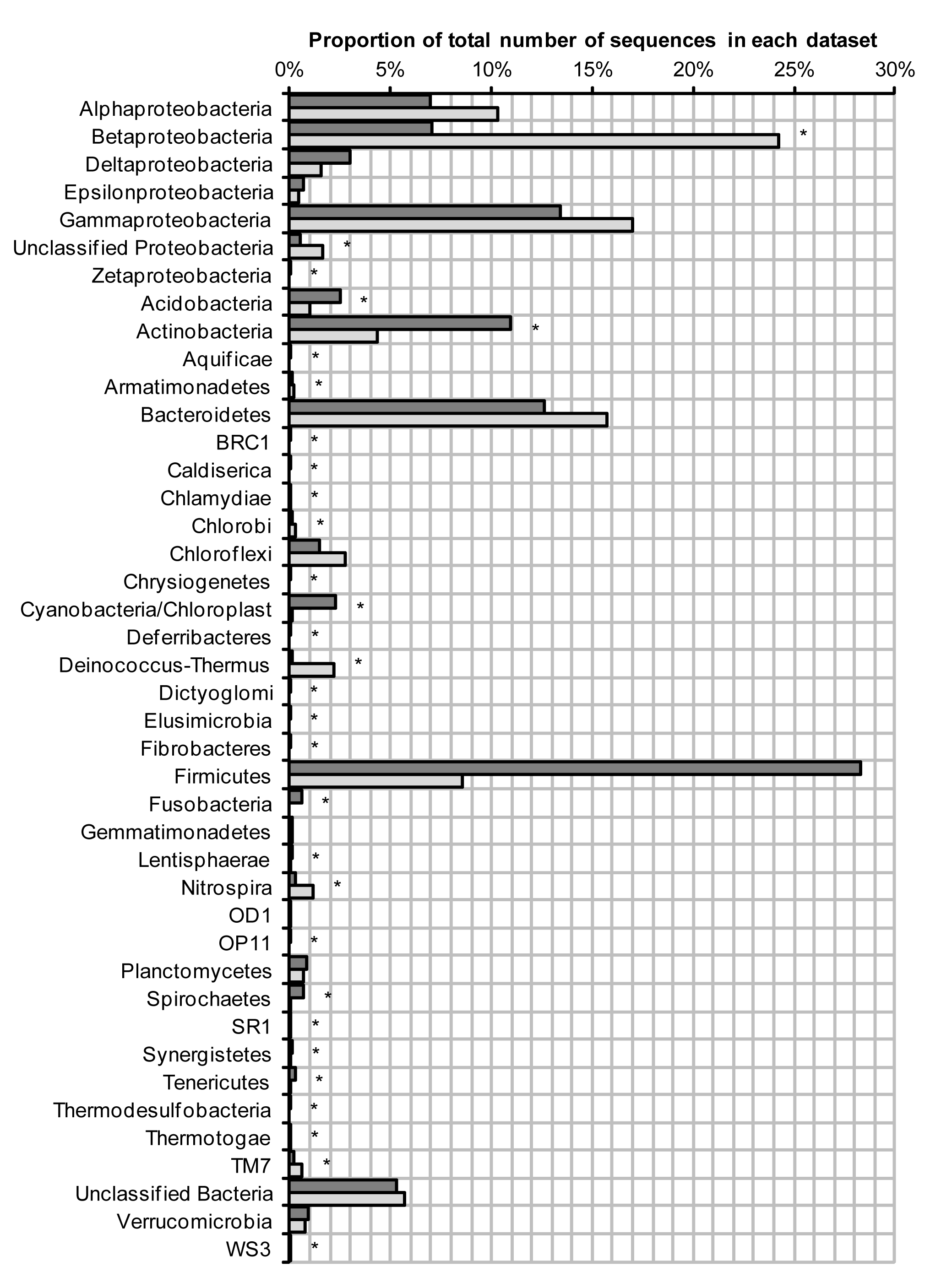

Supplement: Figure S2 — Composition of the sequence databases. Distribution of 2 324 034 sequences in the RDP database (dark gray bars) and 10878 bacterial sequences in the activated sludge subset of the RDP database (Light gray bars). *Phylum or class with a proportion at least twice as large in one of the datasets than in the other. (TIF) [file pone.0076431.s002.tif]

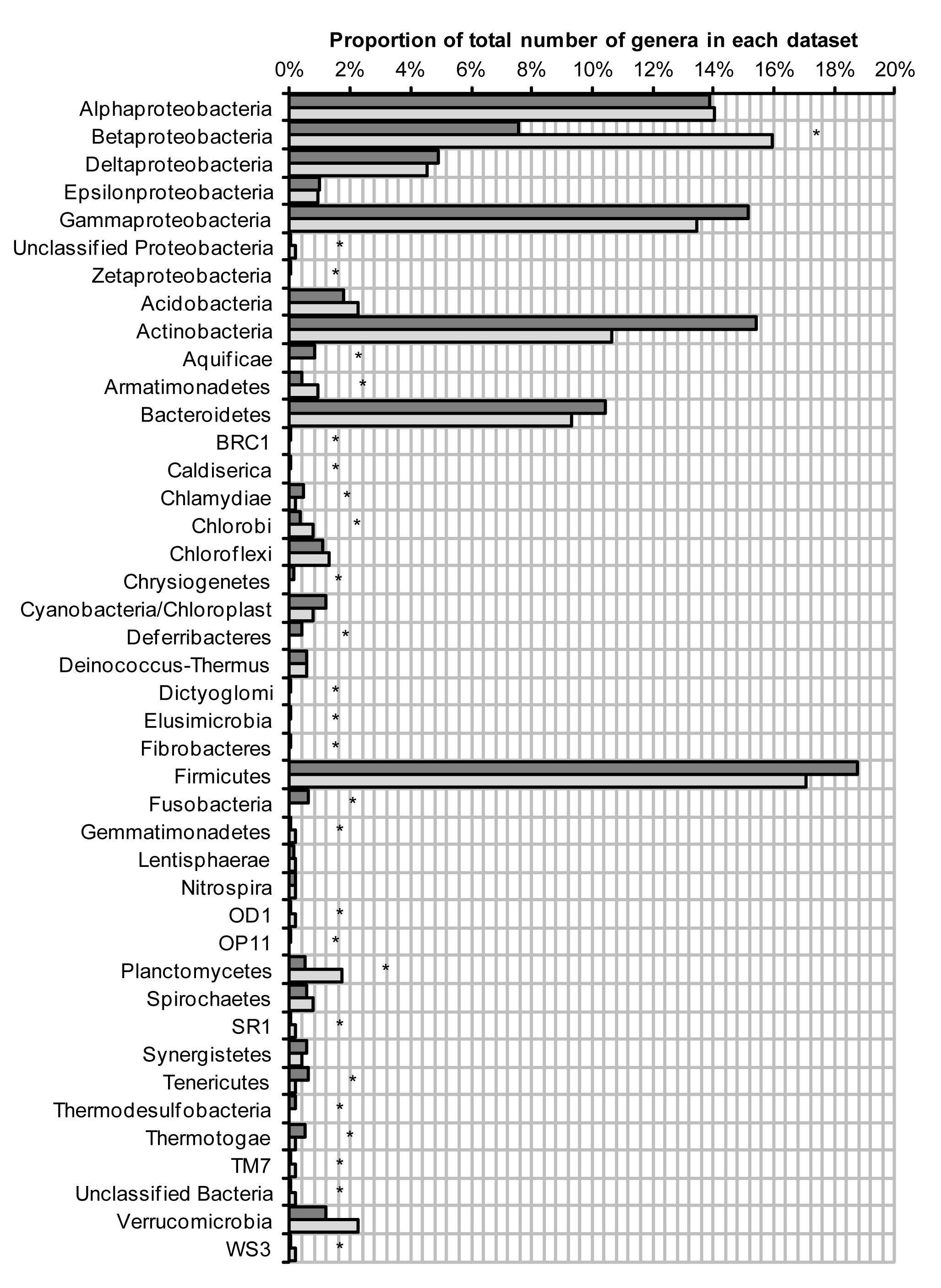

Supplement: Figure S3 — Genus richness of the sequence databases. Number of genera within each taxa expressed as the proportion of the total number of genera in the RDP database (dark gray bars) and in the activated sludge subset of the RDP database (Light gray bars). *Phylum or class with a proportion at least twice as large in one of the datasets than in the other. (TIF) [file pone.0076431.s003.tif]

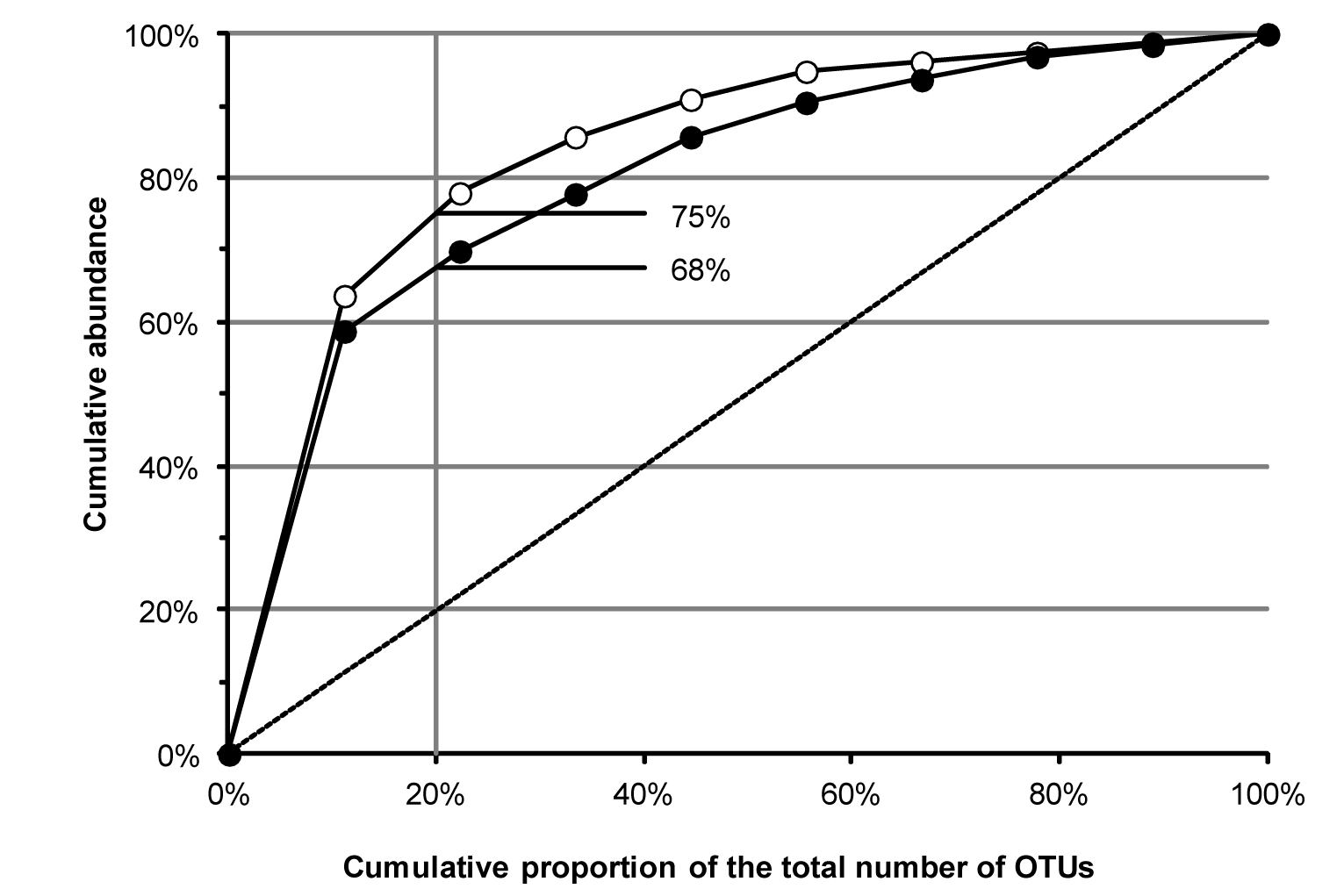

Supplement: Figure S4 — Evenness of the gene libraries. Pareto-Lorenz evenness curves of the 16S rRNA gene libraries generated using 27F&1492R (white circles) and 63F&M1387R (black circles). The sequences were divided in OTUs by phyla (including the Proteobacteria classes), as determined by classification. The Fo index for each sequence set is given. (TIF) [file pone.0076431.s004.tif]
