# Supplementary material for: The Choice of PCR Primers Has Great Impact on Assessments of Bacterial Community Diversity and Dynamics in a Wastewater Treatment Plant
Source: PLoS One. 2013 Oct 1;8(10):e76431. doi: 10.1371/journal.pone.0076431 (PMC3788133; doi:10.1371/journal.pone.0076431)
Supplement: Material S1 — Comments regarding annealing temperature and library size. A supporting discussion about the impact of PCR annealing temperature and library size on the observed difference in composition between the gene library generated with the primer pair 27F&1492R and the library generated with 63F&M1387R. (PDF) [file pone.0076431.s005.pdf]

THE CHOICE OF PCR PRIMERS HAS GREAT IMPACT ON ASSESSMENTS OF BACTERIAL COMMUNITY DIVERSITY AND DYNAMICS IN A WASTEWATER TREATMENT PLANT – SUPPORTING MATERIAL S1

Nils Johan Fredriksson[1]*, Malte Hermansson[2], Britt-Marie Wilén[1]

[1]Department of Civil and Environmental Engineering, Water Environment Technology, Chalmers University of Technology, 412 96 Gothenburg, Sweden

[2] Department of Chemistry and Molecular Biology, University of Gothenburg, Gothenburg, Sweden

*Corresponding author. E-mail: johan.fredriksson@chalmers.se

COMMENTS REGARDING ANNEALING TEMPERATURE AND LIBRARY SIZE

In addition to the primer sequences or the target regions, there are several other factors in the PCR-based analysis of 16S rRNA genes which may affect the observed diversity (e.g. [1]), and this should be taken into account in experimental comparisons of primer pairs. Different annealing temperatures during PCR have been shown to affect the number of observed T-RFs [2,3] or denaturing gradient gel electrophoresis bands [4] in environmental surveys. In this study, the gene libraries were generated with high annealing temperatures in the PCR, 55°C and 60°C, for the 27F&1492R and the 63F&M1387R primer pairs, respectively. A theoretical evaluation suggested that lowering the annealing temperature of the 63F&M1387R primer pair (i.e. allowing more mismatches between primer and template) would increase the similarity of the communities targeted by the two primer pairs (Figure S5). However, T-RFLP analysis of an activated sludge sample showed that decreasing the annealing temperature from 60 to 55 °C did not change the T-RF profiles much. The profiles generated using different annealing temperatures shared all but two T-RFs, each T-RF profile having one unique T-RF (Figure S6).

Another analysis parameter that affects the observed diversity is the gene library size [5]. To evaluate if the small library sizes contributed to the observed differences, the compatibility of the sequences targeted by 27F&1492R with the 63F&M1387R primers was assessed (Figure S5). With one allowed mismatch there were eight sequences in the 27F&1492R library matching the 63F&M1387 primers but only one of these sequences were found in both libraries. Hence, there were seven sequences in the 27F&1492R library that at least theoretically could have been amplified by the 63F&M1387R primers and might have been observed in the 63F&M1387R library if it had been larger. A small part of the difference in composition between the gene libraries can therefore be attributed to the small library sizes. The fact that the small library sizes only explain a small part of the differences between the two primer pairs is supported by the T-RFLP analysis, which is based on all amplified 16S rRNA sequences and not on a small random selection of sequences, as the gene libraries are. The T-RF profiles generated with different primer pairs were distinct, sharing only a few T-RFs (Figure 3). The observed differences between the T-RF profiles were also found to be significant in an analysis of similarity (ANOSIM), indicating that the two primer pairs generated two different sequence sets. As neither small library

size nor high annealing temperatures can explain all of the observed differences between the gene libraries generated by 27F&1492R and 63F&M1387R, we conclude that the difference in composition of the gene libraries is mainly due the difference in targeted populations between the two primer pairs.

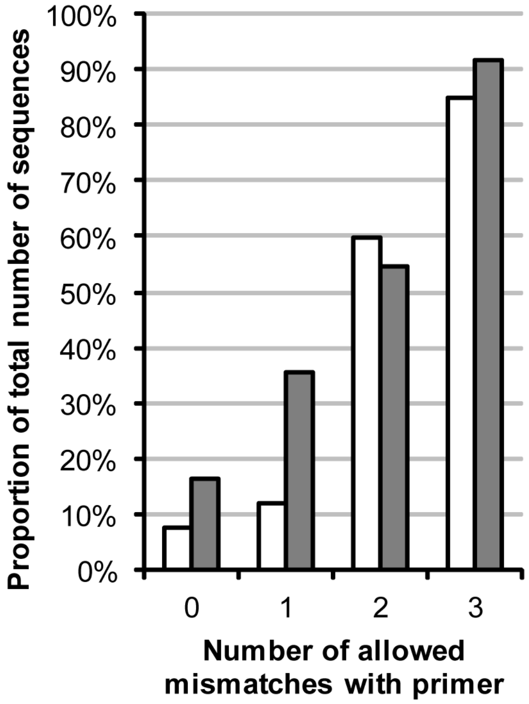

Figure S5. Number of allowed mismatches versus number of sequences targeted by both primer pairs. Proportion of sequences in the 27F&1492R gene library (white columns) and in the RDP 27F&1492R datasets (gray columns) that matches the 63F&M1387R primers.

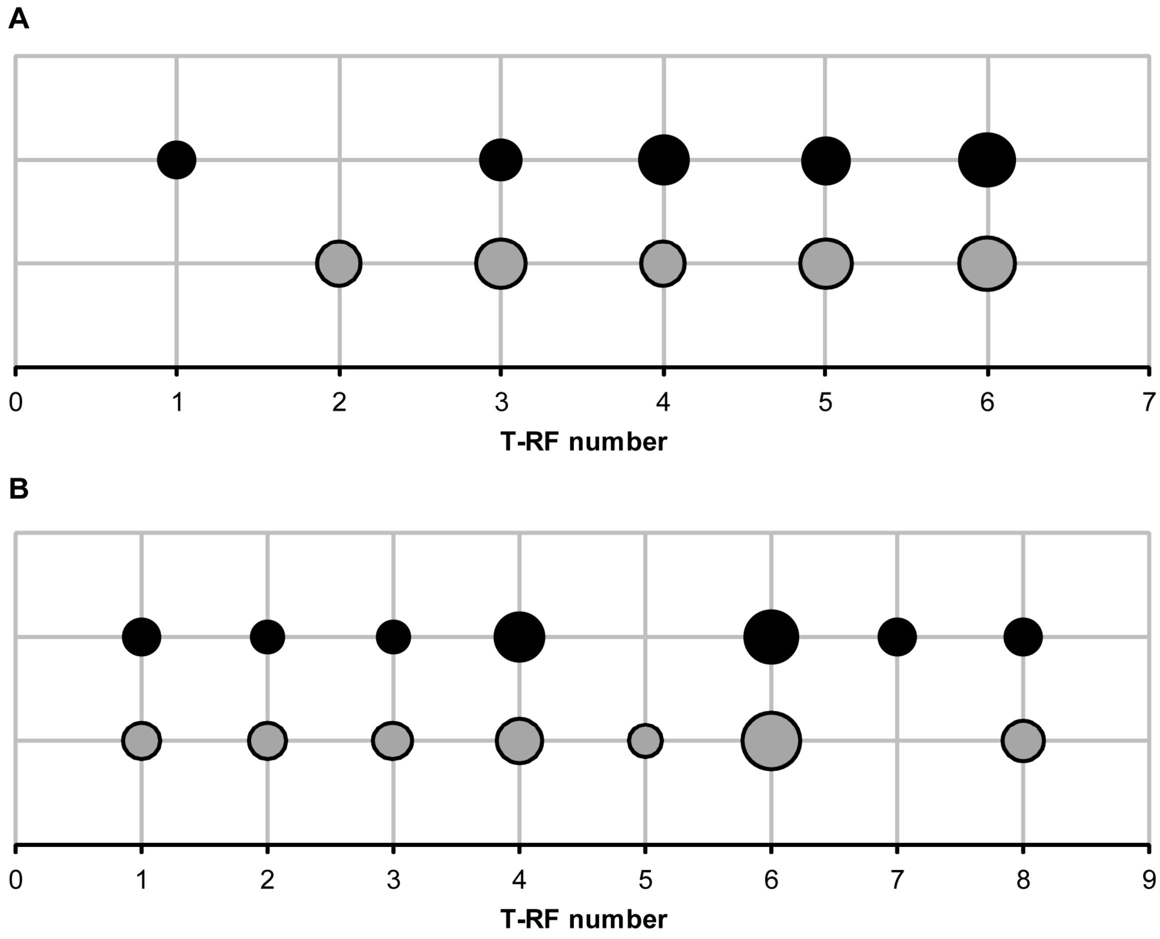

Figure S6. Comparison of T-RF profiles generated with different annealing temperatures. T-RF profiles of an activated sludge sample generated with 63F&M1387R and PCR annealing temperatures 55°C (gray circles) and 60°C (black circles) using restriction enzymes *Hha*I (panel A) and *Rsa*I (panel B). To allow for alignment of the T-RFs, 35 bases was added to the lengths of all T-RFs in the 63F&M1387R profiles. The size of a circle corresponds to the relative abundance of the T-RF, i.e. the peak height divided by the sum of all peak heights in the profile.

References

1. Wintzingerode FV, Göbel UB, Stackebrandt E (1997) Determination of microbial diversity in environmental samples: Pitfalls of PCR-based rRNA analysis. FEMS Microbiology Reviews 21: 213-229.
2. Sipos R, Székely AJ, Palatinszky M, Révész S, Márialigeti K, et al. (2007) Effect of primer mismatch, annealing temperature and PCR cycle number on 16S rRNA gene-targetting bacterial community analysis. FEMS Microbiology Ecology 60: 341-350.
3. Osborn AM, Moore ERB, Timmis KN (2000) An evaluation of terminal-restriction fragment length polymorphism (T-RFLP) analysis for the study of microbial community structure and dynamics. Environmental Microbiology 2: 39-50.
4. Ishii K, Fukui M (2001) Optimization of annealing temperature to reduce bias caused by a primer mismatch in multitemplate PCR. Appl Environ Microbiol 67: 3753-3755.
5. Kemp P, F., Aller J, Y. (2004) Estimating prokaryotic diversity: When are 16S rDNA libraries large enough? Limnol Oceanogr Methods 2: 114-125.
