# Supplementary material for: The Choice of PCR Primers Has Great Impact on Assessments of Bacterial Community Diversity and Dynamics in a Wastewater Treatment Plant
Source: PLoS One. 2013 Oct 1;8(10):e76431. doi: 10.1371/journal.pone.0076431 (PMC3788133; doi:10.1371/journal.pone.0076431)
Supplement: Table S1 — Number of 2 441 787 high quality sequences in the RDP database matching different primers. (PDF) [file pone.0076431.s006.pdf]

Table S1. Number of 2 441 787 high quality sequences in the RDP database matching different primers.

Number of mismatches allowed	0	1	2	3
**27F&1492R**	35926	57086	75361	119395
**27F&1492R but not 63F&M1387R**	30051	36813	34059	14477
**63F&1387R**	52363	152042	259616	450640
**63F&M1387R**	52740	153122	263574	453724
**63F&M1387R but not 27F&1492R**	46865	132849	222272	348806
